# Rapid prediction of vancomycin-resistant *Enterococcus faecium* using MALDI-TOF mass spectrometry and machine learning

**DOI:** 10.3389/fmicb.2026.1789841

**Published:** 2026-06-16

**Authors:** Yanrui Sun, Xin Chen, Aiping Hu, Yuwei Li, Guangping Li, Lijie Zhao, Congcong Wang

**Affiliations:** 1 Department of Clinical Laboratory, Tangshan Gongren Hospital, Tangshan, China; 2 Department of Laboratory Medicine, Foshan Fosun Chancheng Hospital, Foshan, China; 3 Department of Gastroenterology, Tangshan Gongren Hospital, Tangshan, China; 4 Department of Clinical Laboratory, Tangshan Nanhu Hospital, Tangshan, China; 5 Department of Clinical Laboratory, Laoting County Hospital, Tangshan, China; 6 Department of Clinical Laboratory, Hebei Provincial Hospital of Chinese Medicine, Shijiazhuang, China

**Keywords:** *Enterococcus faecium*, machine learning, MALDI-TOF MS, multicenter data integration, vancomycin resistance

## Abstract

**Background:**

Vancomycin-resistant *Enterococcus faecium* (VRE*fm*) is a World Health Organization priority pathogen, yet conventional phenotypic susceptibility testing requires up to 72 h, delaying targeted antimicrobial therapy. This study aimed to develop an interpretable and rapid machine learning classifier to predict VRE*fm* using matrix-assisted laser desorption/ionization time-of-flight mass spectrometry (MALDI-TOF MS) spectra.

**Methods:**

We retrospectively analyzed 268 clinical *E. faecium* isolates (100 VRE*fm* and 168 vancomycin-susceptible *E. faecium*) from Tangshan Gongren Hospital, a tertiary hospital in northern China (2020–2025). Spectra were preprocessed with smoothing, baseline removal, and 5-Da binning (range 2,000–20,000 Da). Extreme gradient boosting recursive feature elimination selected 40 discriminative mass-to-charge ratio features. To enhance model generalizability across diverse bacterial lineages, the training set combined local isolates from 2020 to 2024 with the multi-center DRIAMS repository (subsets A–D). Five classifiers were trained under stratified 5-fold cross-validation and temporally validated on the independent 2025 local isolates. Model discrimination, calibration, and clinical utility were evaluated using the area under the receiver operating characteristic curve (AUROC), Brier score, and decision curve analysis.

**Results:**

The k-nearest neighbors classifier achieved optimal temporal validation performance (area under the receiver operating characteristic curve 0.90, F1 score 0.683, Brier score 0.185). Hybrid training configurations combining local data with pooled DRIAMS subsets retained clinically useful discrimination (AUROC > 0.80), whereas external-only models performed at chance level (AUROC ≈ 0.50).

**Conclusion:**

MALDI-TOF MS combined with machine learning enables rapid, interpretable prediction of vancomycin resistance in *E. faecium*. A hybrid local–multi-center training strategy offers a pragmatic solution for laboratories with limited local sample availability, facilitating clinical deployment of spectral-based antimicrobial resistance surveillance.

## Graphical abstract

**Figure:**
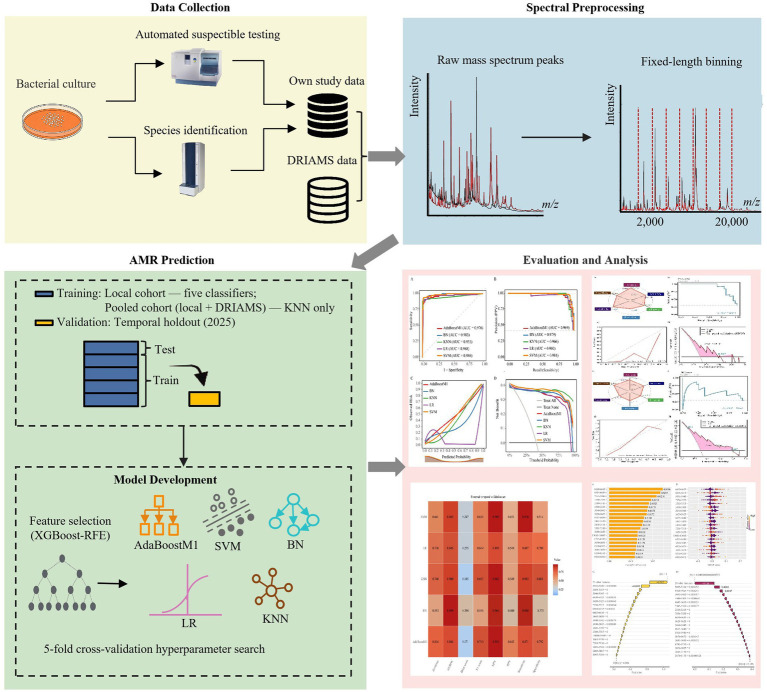
Integrated workflow and key findings.

## 1 Introduction

*Enterococcus* spp. are important opportunistic pathogens in hospital-acquired infections, capable of causing severe conditions such as urinary tract infections, abdominal infections, bloodstream infections, and endocarditis (García-Solache and Rice, 2019; Reynolds et al., 2021). This genus is naturally resistant to various antibiotics, including cephalosporins, polymyxins, and macrolides (Clinical and Laboratory Standards Institute, 2019; EUCAST, 2019). The clinical use of vancomycin has further selected for vancomycin-resistant enterococci (VRE) (Top et al., 2007; García-Solache and Rice, 2019; Gök et al., 2020), among which vancomycin-resistant *Enterococcus faecium* (VRE*fm*) accounts for 60% of VRE cases (Wisplinghoff et al., 2004; Fiore et al., 2019; Stagliano et al., 2021). Treatment options for VRE infections are limited, leading to prolonged hospital stays; for example, the average length of hospitalization for patients with VRE bloodstream infections is 35 ± 22.5 days (da Silva et al., 2014). The mortality rate of hospitalized patients with VRE ranges from 23 to 65% (Somily et al., 2016; Tripathi et al., 2016; Hughes et al., 2019). The World Health Organization has listed VRE as a priority pathogen and designated it as requiring new treatment methods by 2024 (Sati et al., 2025). Early identification of the pathogen and its resistance profile can reduce 30-day mortality among hospitalized patients (Forrest et al., 2008). Currently, antibiotic susceptibility testing (AST) is the primary method used to assess bacterial resistance; however, the time from sample collection to resistance reporting can be as long as 72 h. This delay often leads clinicians to use empirical or broad-spectrum antibiotics early in treatment, increasing unnecessary antibiotic exposure and resistance selection pressure. Therefore, there is an urgent need to develop faster and more accurate resistance detection methods that can be directly applied in clinical settings to guide early treatment and improve patient outcomes (Gajic et al., 2022).

In recent years, matrix-assisted laser desorption/ionization time-of-flight mass spectrometry (MALDI-TOF MS) has become a mainstream tool in clinical microbiology laboratories worldwide due to its high throughput, rapidity, and cost-effectiveness (Pizzato et al., 2022). It can generate spectra containing hundreds of ion peaks from a single colony within seconds to minutes. Some studies have used these spectra to predict resistance by applying software protocols (Xiao et al., 2014; Chen et al., 2017). However, methods based on spectrum averages often identify only the most prominent differential peaks, potentially missing sub-significant signals associated with complex phenotypes such as multigenic resistance regulation. These methods are also susceptible to bias from outliers, which may lead to inconsistent results (Hrabák et al., 2013). This limitation restricts the clinical application of such approaches. Machine learning has the capability to extract patterns, reveal associations, and establish predictive relationships from complex datasets (Poirion et al., 2021) and has been applied across various fields in life sciences (Cho and Kang, 2020). Machine learning models can handle large-scale, high-dimensional data to extract underlying biological signals, thereby improving the accuracy and speed of resistance prediction. Additionally, the extraction of supplementary information directly from acquired MALDI-TOF MS enables the inference of antimicrobial susceptibility (Kim et al., 2019). By systematically integrating these advantages, it is possible not only to overcome the time limitations of traditional detection methods but also to provide scientific evidence for clinical drug selection and infection control.

Several studies have employed machine learning methods to analyze MALDI-TOF MS for rapid identification and characterization of VRE*fm*. These studies have demonstrated the feasibility of distinguishing VRE*fm* from vancomycin-susceptible *Enterococcus faecium* (VSE*fm*) based on spectral features or whole-spectrum learning (Griffin et al., 2012; Wang et al., 2021, 2022). However, single-center studies from diverse geographic settings are needed to incrementally expand the currently limited evidence base for machine learning-enhanced VRE*fm* detection by MALDI-TOF MS.

We aimed to develop a machine learning framework utilizing MALDI-TOF MS for rapid prediction of VRE*fm*. Using local cohort spectral data, we employed extreme gradient boosting recursive feature elimination (XGBoost-RFE) to identify discriminative feature peaks, and constructed and compared five distinct classifiers. Rigorous temporal validation was subsequently performed on local isolates. To ensure broad generalizability across diverse bacterial lineages, we further incorporated the publicly available DRIAMS database (subsets A–D; Diagnostic Resistance Information based on Antimicrobial Susceptibility Testing and Mass Spectrometry) as a multi-center training resource, thereby enhancing model robustness for prospective clinical deployment.

## 2 Materials and methods

### 2.1 Data sources and approval

Data were obtained from two sources. The development cohort was derived from the Clinical Microbiology Laboratory of Tangshan Gongren Hospital, Hebei Province (January 2020–December 2025). For strains repeatedly detected during the same hospitalization, only the first detected strain was included in the analysis. Finally, 268 strains were obtained, including 100 strains of VRE*fm* and 168 strains of VSE*fm*. To enhance model transferability across heterogeneous strain backgrounds, we leveraged the publicly available DRIAMS database,^1^ which was developed by Weis et al. (2022). This repository contains over 300,000 MALDI-TOF mass spectra of clinical microorganisms collected from four clinical microbiologic laboratories (DRIAMS A–D) in Switzerland between 2015 and 2018. All spectral profiles were acquired using the Microflex Biotyper System (Bruker Daltonics, Bremen, Germany). From this repository, we extracted *E. faecium* isolates with confirmed vancomycin phenotypes (VRE*fm* and VSE*fm*) and their mass spectral profiles to construct a pooled training set. This study was approved by the Ethics Committee of Tangshan Gongren Hospital (Approval No. GRYY-LL-KJ2021-K28). This research adhered to the guidelines for Transparent Reporting of Multivariable Prediction Models Incorporating Artificial Intelligence in Individual Prognosis or Diagnosis (TRIPOD+AI) (Collins et al., 2024).

### 2.2 Species identification and vancomycin susceptibility testing

For the local isolates, species identification and AST were performed using the VITEK II automated microbiology identification system (bioMérieux, Marcy-l’Étoile, France) according to the manufacturer’s instructions (Hegstad et al., 2014; Bobenchik et al., 2015). Results were interpreted according to the CLSI M100 breakpoint standards (Clinical and Laboratory Standards Institute, 2019). For VRE*fm*, further confirmation was conducted using the E-test method (bioMérieux, France).

### 2.3 Sample preparation

Isolates were recovered from cryopreserved storage (−80 °C) via sequential subculturing on Columbia blood agar. Spectra for the 2020–2024 training set were acquired following 24-h incubation, whereas those for the 2025 temporal validation set were acquired following both 24- and 48-h incubation. Subsequently, spectra were acquired using MALDI-TOF MS (Bruker Daltonik GmbH, Bremen, Germany) according to manufacturer specifications (MBT Compass v4.1). The procedure commenced as follows: a single isolated colony was aseptically transferred into 300 μL of molecular-grade water (Sigma-Aldrich, St. Louis, MO, USA) contained in a 1.5 mL microcentrifuge tube. Homogenization was achieved through at least 10 careful pipetting cycles followed by vortexing at 2,000 rpm for 1 min. Cell fixation was then initiated by adding 900 μL of absolute ethanol (≥99.8% purity; Sigma-Aldrich, St. Louis, MO, USA), followed by vortexing for 1 min and centrifugation (13,000 × *g*, 2 min). The supernatant was carefully removed, and the pellet was washed a second time with ethanol to eliminate potential contaminants. Residual ethanol was allowed to evaporate under ambient conditions. Subsequently, the bacterial pellet was resuspended in 50 μL of 70% formic acid (Sigma-Aldrich, St. Louis, MO, USA) with vortexing at 2,500 rpm for 30 s. Then, 50 μL of HPLC-grade acetonitrile (Sigma-Aldrich, St. Louis, MO, USA) was added, and the mixture was vortexed again to ensure thorough mixing. The cell lysate was clarified by centrifugation (13,000 × *g*, 2 min) to pellet debris. A 1 μL aliquot of the resultant supernatant was spotted onto a precleaned MALDI target plate (MSP 96 target; Bruker Daltonics, Bremen, Germany). After air-drying at room temperature, the sample was overlaid with 1 μL of a 1% α-cyano-4-hydroxycinnamic acid (HCCA) matrix solution containing 2.5% trifluoroacetic acid in 50% acetonitrile (Sigma-Aldrich, St. Louis, MO, USA). Finally, the sample was left to crystallize fully at room temperature before spectral acquisition.

### 2.4 Data collection and processing

Mass spectrometry analysis was performed in linear positive ion mode with an acceleration voltage set to 20 kV. The experiment used a nitrogen laser (60 Hz repetition rate), accumulating signals from 240 laser pulses for each sample point, with a mass range of 2,000–20,000 Da. To ensure data accuracy, Bruker Daltonics bacterial detection standards were used as external references for spectral calibration. Bacterial identification was completed using the Biotyper v3.1 system where identification results with a log score ≥2.0 were considered valid. Raw spectra from local isolates were imported into flexAnalysis v3.4 software (Bruker Daltonics) for initial preprocessing. Both the local and DRIAMS datasets were subsequently internally calibrated using the conserved peak at *m/z* 4,429 (Wang et al., 2021). The same preprocessing pipeline was applied to both the local and DRIAMS datasets, based on the DRIAMS protocol (Weis et al., 2022) with the binning interval adjusted from 3 Da to 5 Da. Preprocessing was performed using the R package MaldiQuant (Gibb and Strimmer, 2012) v1.19. The steps included applying a square root transformation to the measured intensities to stabilize variance; using the Savitzky–Golay algorithm with a half—window size of 10 for spectral smoothing; performing 20 iterations of the SNIP algorithm to remove baseline estimates; calibrating intensities using total ion current (TIC); and trimming the spectra to values within the range of 2,000–20,000 Da. After preprocessing, a total of 371 spectral files were generated, with each spectrum containing intensity and *m/z* values. Due to variability in *m/z* and intensity recording caused by resolution and the acquisition process, the dimensionality of each sample may vary, leading to different numbers of data points. Additionally, measurements across samples are typically non-uniformly spaced. Because the machine-learning approaches employed in this study require fixed-length input vectors, we discretized the *m/z* axis using 5 Da bins, a choice informed by preliminary analyses and previous studies (Mather et al., 2016; Wang et al., 2021; Gao et al., 2024; López-Cortés et al., 2025). The *m/z* range 2,000–20,000 Da was therefore divided into 3,600 consecutive 5 Da bins, and intensities within each bin were summed to yield fixed-dimensional feature vectors for subsequent model development.

### 2.5 Feature selection and model development

**Figure 1:**
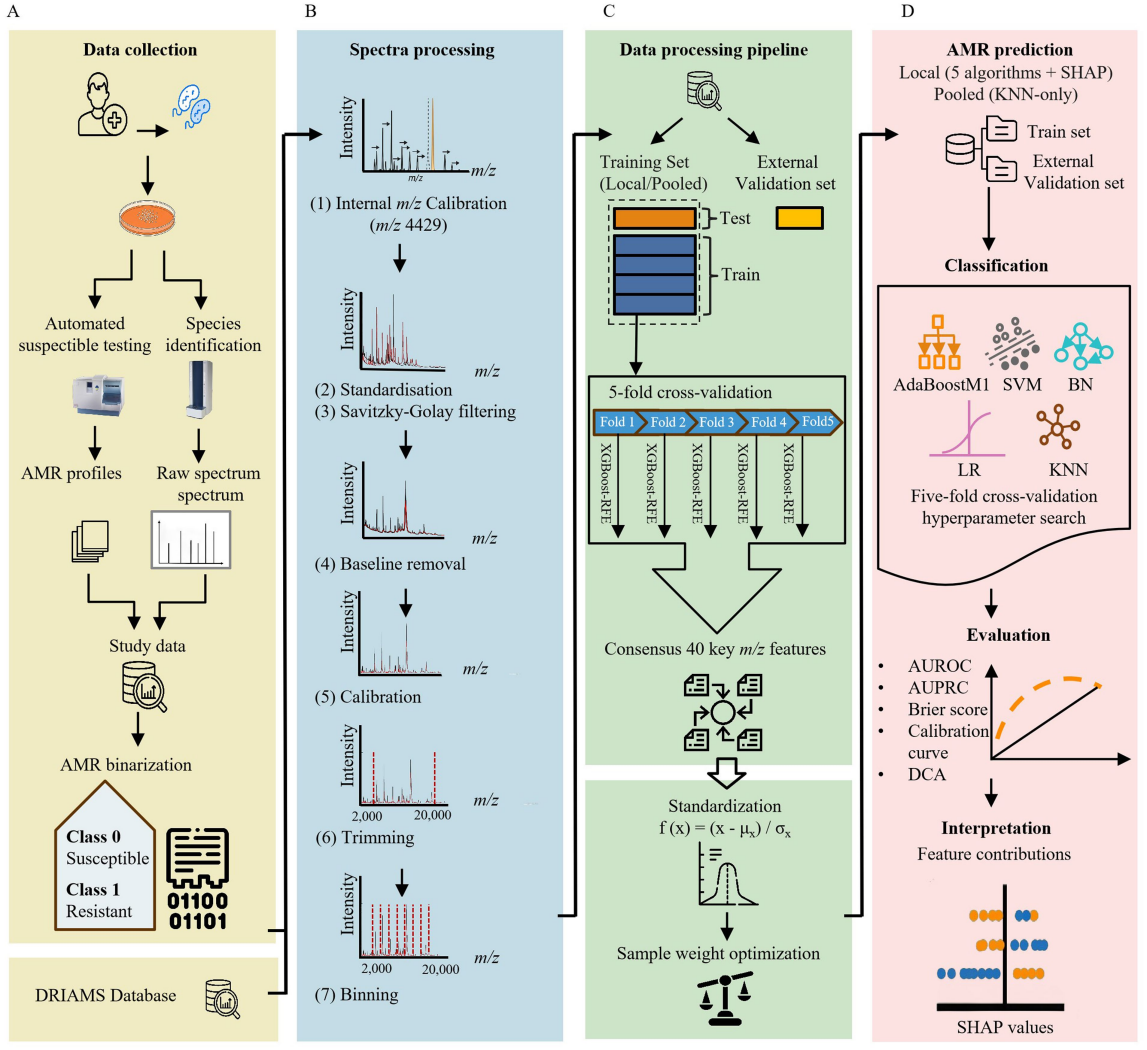
Workflow of the machine learning-based MALDI-TOF MS pipeline for predicting vancomycin resistance in *Enterococcus faecium*. **(A)** Data collection and antimicrobial resistance (AMR) binarization. Clinical isolates underwent automated susceptibility testing and MALDI-TOF MS species identification, yielding AMR profiles and raw mass spectra that were integrated and binarized into susceptible (Class 0) and resistant (Class 1) phenotypes. The public DRIAMS database was incorporated for spatial external validation. **(B)** Spectral preprocessing. Raw spectra underwent internal *m/z* calibration (*m/z* 4,429), standardization, Savitzky–Golay smoothing, baseline removal, intensity calibration, trimming (2,000–20,000 Da), and binning. **(C)** Model development pipeline. Training sets (local or pooled) were subjected to 5-fold cross-validation. XGBoost-based recursive feature elimination (XGBoost-RFE) was performed in each fold, producing a consensus set of 40 key *m/z* features. Features were standardized (*z*-score) and sample weights were optimized. **(D)** Prediction, evaluation, and interpretation. For the local dataset, five algorithms—AdaBoostM1 (adaptive boosting algorithm M1), support vector machine (SVM), Bayesian network (BN), logistic regression (LR), and k-nearest neighbors (KNN)—underwent 5-fold cross-validation with hyperparameter search, followed by temporal external validation and SHAP-based interpretability analysis. The pooled dataset was evaluated with KNN-only for spatial external validation. Performance was assessed using the area under the receiver operating characteristic curve (AUROC), area under the precision-recall curve (AUPRC), Brier score, calibration curves, and decision curve analysis (DCA). Source: https://www.magnific.com/icons.

To develop and validate a machine-learning model to discriminate VRE*fm* from VSE*fm*, we followed the workflow shown in Figures 1C,D. This workflow was designed to rigorously prevent data leakage and ensure an unbiased evaluation.

Spectral data collected after 24 h of incubation in 2020–2024 were used to train the model. The 2025 dataset, comprising spectra from the same isolate collection, served as a temporal external validation set. The 24-h 2025 spectra were used to assess prospective performance under standard incubation conditions, while the 48-h spectra evaluated the model’s robustness to extended incubation. Spatial external validation was conducted using the DRIAMS-A dataset (2016–2018). To determine optimal model configurations, 5-fold cross-validation was subsequently performed solely on training data. Within this framework, iterative feature importance ranking and selection were conducted using the XGBoost-RFE algorithm. Specifically, within each fold, XGBoost-RFE utilized only the corresponding training partition to identify significant features. Models trained with these selected features were then evaluated exclusively against the validation subset of that fold. This procedure was repeated across all folds. Based on cross-fold stability assessment, a robust subset of 40 key *m/z* features was ultimately selected for downstream modelling.

For antimicrobial resistance classification, five machine-learning algorithms were selected based on prior MALDI-TOF MS studies: adaptive boosting (AdaBoostM1), Bayesian network (BN), k-nearest neighbors (KNN), logistic regression (LR), and support vector machine (SVM). Hyperparameter tuning employed a hybrid strategy: Bayesian optimization to efficiently survey the parameter space, followed by targeted manual refinement to finalize model configurations. The optimal hyperparameter set for each algorithm was selected based on area under the receiver operating characteristic curve (AUROC) from stratified 5-fold cross-validation. After feature selection, each model was retrained on the entire training dataset, and the resulting classifiers were used for local prediction tasks. Model training was conducted exclusively on the training set; features were *z*-score standardized, and class weights were applied to mitigate class imbalance. Optimal hyperparameters for the KNN classifier are detailed in Supplementary Table 1.

### 2.6 Cross-center validation strategy

To broaden coverage across diverse bacterial lineages, we trained the optimal KNN classifier under four distinct configurations: (i) DRIAMS-A; (ii) local training set combined with DRIAMS-A; (iii) pooled DRIAMS subsets (A–D); and (iv) local training set combined with pooled DRIAMS subsets (A–D). All candidate models were independently assessed on the local 24-h temporal validation set. Notably, DRIAMS-A data from 2015 were excluded as they lacked vancomycin-resistant strains, which would have compromised the binary classification objective.

### 2.7 Evaluation metrics

To illustrate the classification performance of each model, we constructed confusion matrices. A confusion matrix is a tabular evaluation tool that summarizes the correspondence between predicted and true labels, where rows represent true classes and columns represent predicted classes. From these matrices, we extracted four core elements: true positives (TP), true negatives (TN), false positives (FP), and false negatives (FN). Based on these counts, we computed comprehensive performance metrics, including accuracy, recall (sensitivity), F1 score, AUROC, and area under the precision–recall curve (AUPRC), to characterize model behavior systematically. To balance discrimination and calibration during model selection, we adopted a composite metric, *AB*_score_ (Li et al., 2026):


$$AB_{\text{score}}=\alpha \cdot \bar{\;\text{AUROC}}+\alpha \cdot (1-\bar{\text{Brier score}}),$$

Where *α* is a weighting coefficient (*α* = 0.5 to weight discrimination and calibration equally). The term (1 − $\bar{\text{Brier score}}$ ) aligns the Brier-derived component with the positive direction of AUROC so that larger *AB*_score_ values consistently indicate superior performance. $\bar{\text{AUROC}}$ and $\bar{\text{Brier score}}$ are the arithmetic means of AUROC and Brier scores computed across internal cross-validation folds and the temporal external validation set. This composite measure mitigates the risk of selecting models that exhibit high discrimination but poor calibration by incorporating both aspects into the selection criterion.

For the final model, discrimination was evaluated using AUROC and AUPRC, calibration using the Brier score and calibration plots (Stevens and Poppe, 2020), and clinical utility using decision curve analysis (DCA) across a range of probability thresholds (Vickers and Elkin, 2006). These complementary evaluations ensured selection of a model that provides both reliable ranking and well-calibrated probability estimates for potential clinical application.

### 2.8 Shapley-based feature contribution analysis

Based on the principles of game theory, SHAP (SHapley Additive exPlanations) allocates the contributions of features to model predictions, providing consistent and fair model explanations (Bifarin, 2023). For single-sample interpretation, we visualized SHAP values, which represent the magnitude and direction of individual feature contributions, using SHAP waterfall plots from the R package shapviz (version 0.9.6). These plots illustrate the decomposition of the prediction from the base value to the final output by sequentially accumulating positive and negative feature contributions for individual VRE strains.

### 2.9 Statistical analysis

Model performance on the local and pooled training sets was assessed via a 5-fold cross-validation. Statistical analyses were carried out using R software (version 4.2.2, The R Foundation, http://www.R-project.org) and Free Statistics software version 2.4.0.

## 3 Results

### 3.1 Clinical mass spectrometry database

From January 2020–December 2025, we retrospectively identified and included 268 clinical *E. faecium* isolates (100 VRE*fm* and 168 VSE*fm*) from our local microbiology laboratory. Of these, 165 isolates (collected 2020–2024) constituted the training set, and 103 isolates (collected in 2025; 31 VRE*fm* and 72 VSE*fm*) served as the temporal external validation set. A key advantage of the MALDI-TOF MS workflow is its ability to deliver rapid species identification within 24 h from isolate recovery, substantially accelerating the diagnostic timeline compared with AST, as demonstrated in the 2025 temporal validation cohort (Supplementary Figure 1). We additionally obtained mass spectrometry data and associated vancomycin phenotypic profiles for *E. faecium* from the DRIAMS repository (subsets A–D). The detailed workflow is shown in Figure 1A.

### 3.2 Machine learning approaches for predicting antibiotic resistance

**Figure 2:**
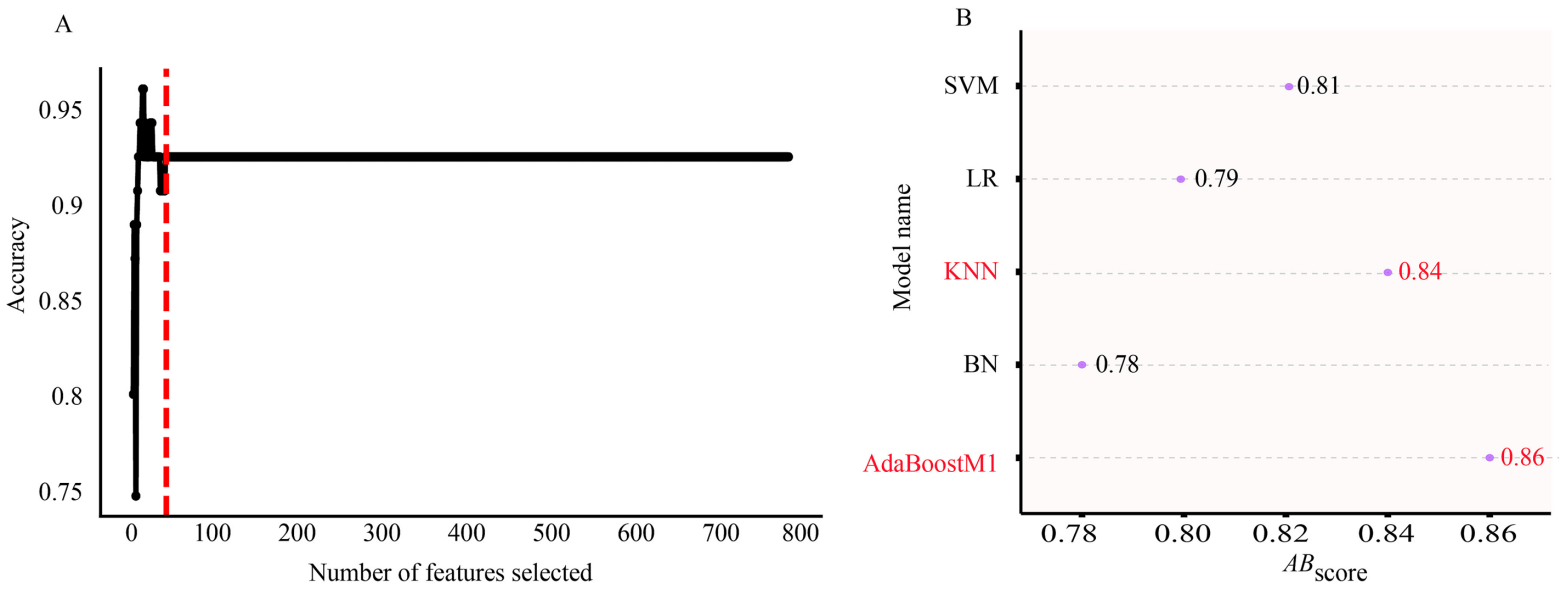
Feature selection and algorithm comparison for predicting vancomycin resistance in *Enterococcus faecium*. **(A)** Accuracy trajectory during XGBoost-based recursive feature elimination (XGBoost-RFE) across 5-fold cross-validation. The dashed red line indicates the selection of 40 key *m/z* features, after which model accuracy plateaued, defining the optimal feature subset for downstream classification. **(B)** Comparative performance of five machine learning algorithms on the temporal external validation set, ranked by the composite metric *AB*_score_. Shown are adaptive boosting algorithm M1(AdaBoostM1), k-nearest neighbors (KNN), support vector machine (SVM), logistic regression (LR), and Bayesian network (BN).

To ensure harmonization across cohorts, mass spectra from both the local dataset and the DRIAMS multi-center repository were uniformly processed into 5-Da intervals (range: 2,000–20,000 Da), yielding standardized 3,600-dimensional feature vectors per sample. This bin size was selected to balance spectral resolution with robustness against instrumental mass drift across heterogeneous acquisition settings. Specifically, 5 Da is sufficiently narrow to resolve adjacent peaks while being wide enough to accommodate minor *m/z* variations across replicate measurements. Feature selection was performed using XGBoost-RFE, ranking mass peaks by their importance scores. As shown in Figure 2A, classification accuracy increased with the number of selected features and eventually plateaued; the top 40 predictive mass peaks (Supplementary Table 2) were thus retained for model development. This trend was consistent across all machine learning algorithms evaluated. We constructed five vancomycin resistance classification models, including LR, SVM, BN, AdaBoostM1, and KNN. Data from 2020 to 2024 were used for model training with 5-fold cross-validation, while 2025 data served as the external temporal validation set. The models’ discrimination, calibration and clinical utility were comprehensively evaluated via AUROC, AUPRC, calibration curves, and DCA, while predictive performance was assessed using accuracy, Brier scores, F1 score and other standard metrics. To evaluate the incremental value of multi-center data integration, we compared the baseline model trained on local 2020–2024 data against enhanced models trained on pooled datasets integrating local 2020–2024 data with the DRIAMS multi-center repository. All configurations were then validated on the independent local 2025 temporal hold-out set. The comprehensive workflow of this study, encompassing data collection, spectral processing, model development, and AMR prediction, is depicted in Figure 1.

### 3.3 Performance comparison of five predictive models

**Figure 3:**
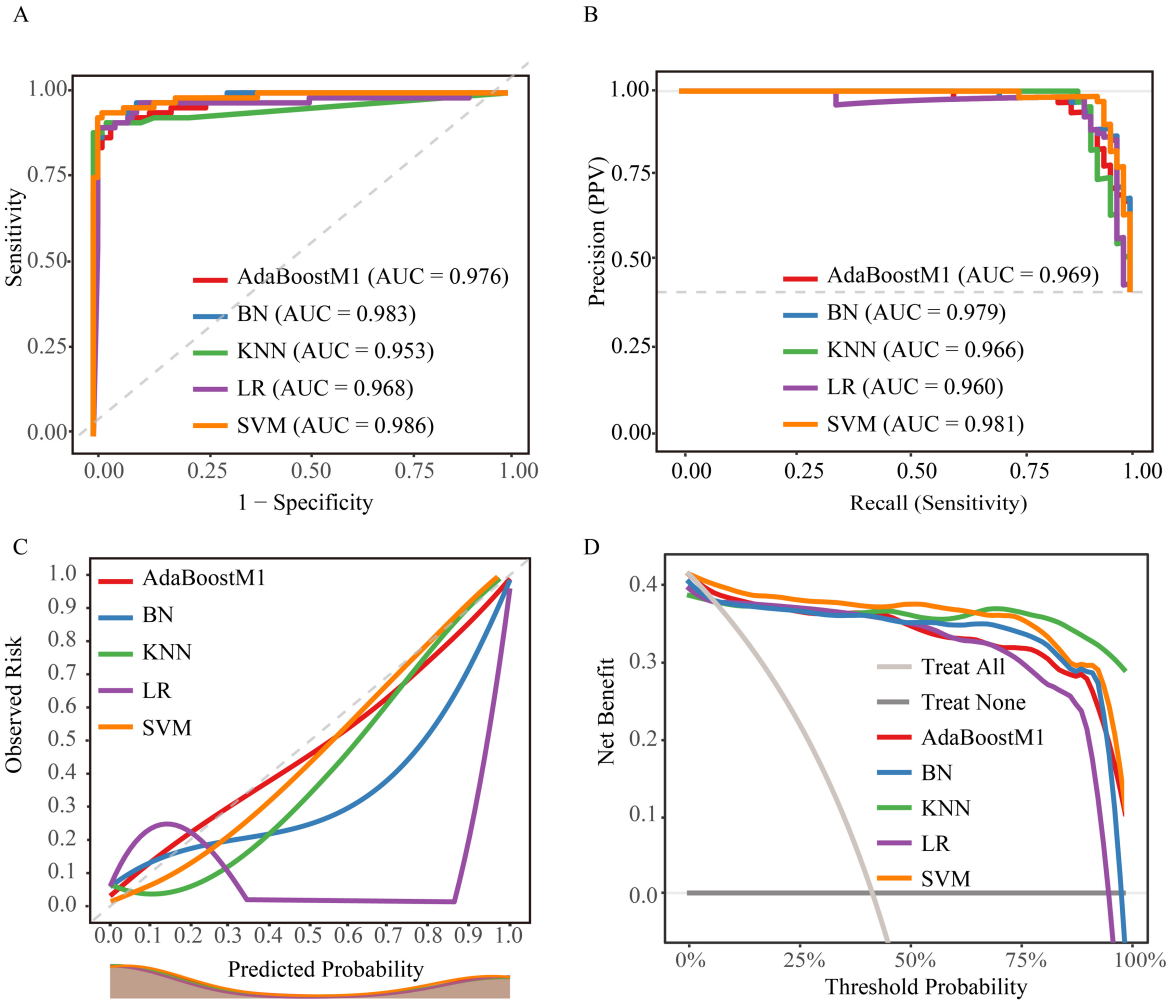
Comprehensive performance evaluation of machine learning classifiers on the training set using 5-fold cross-validation. **(A)** Receiver operating characteristic (ROC) curves for adaptive boosting algorithm M1 (AdaBoostM1), Bayesian network (BN), k-nearest neighbors (KNN), logistic regression (LR), and support vector machine (SVM). **(B)** Precision-recall curves showing positive predictive value (PPV) versus recall (sensitivity) for the five algorithms. **(C)** Calibration plots displaying the relationship between predicted probability and observed risk. **(D)** Decision curve analysis (DCA) comparing the net benefit of each classifier against treat-all and treat-none strategies across threshold probabilities.

**Figure 4:**
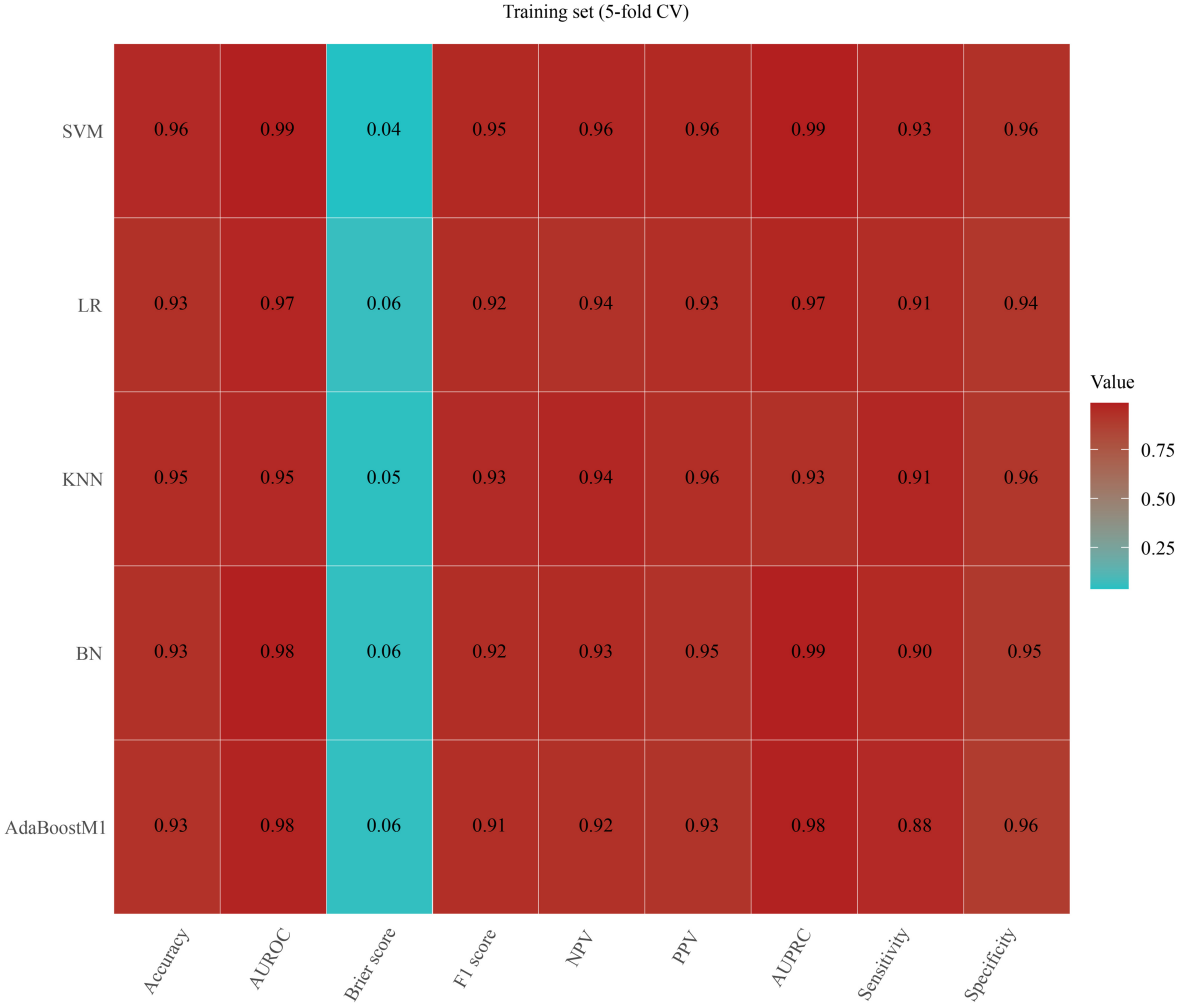
Heatmap of machine learning model performance metrics on the training set (5-fold CV). The heatmap displays the performance of five models (AdaBoostM1, BN, KNN, LR, and SVM) across nine key evaluation metrics. The color gradient (from cyan to red) represents the magnitude of the metric values, with the color bar on the right denoting the value scale. AUROC, area under the receiver operating characteristic curve; AUPRC, area under the precision-recall curve; AdaBoostM1, adaptive boosting algorithm M1; BN, Bayesian network; KNN, k-nearest neighbors; LR, logistic regression; SVM, support vector machine; NPV, negative predictive value; PPV, positive predictive value; CV, cross-validation.

**Figure 5:**
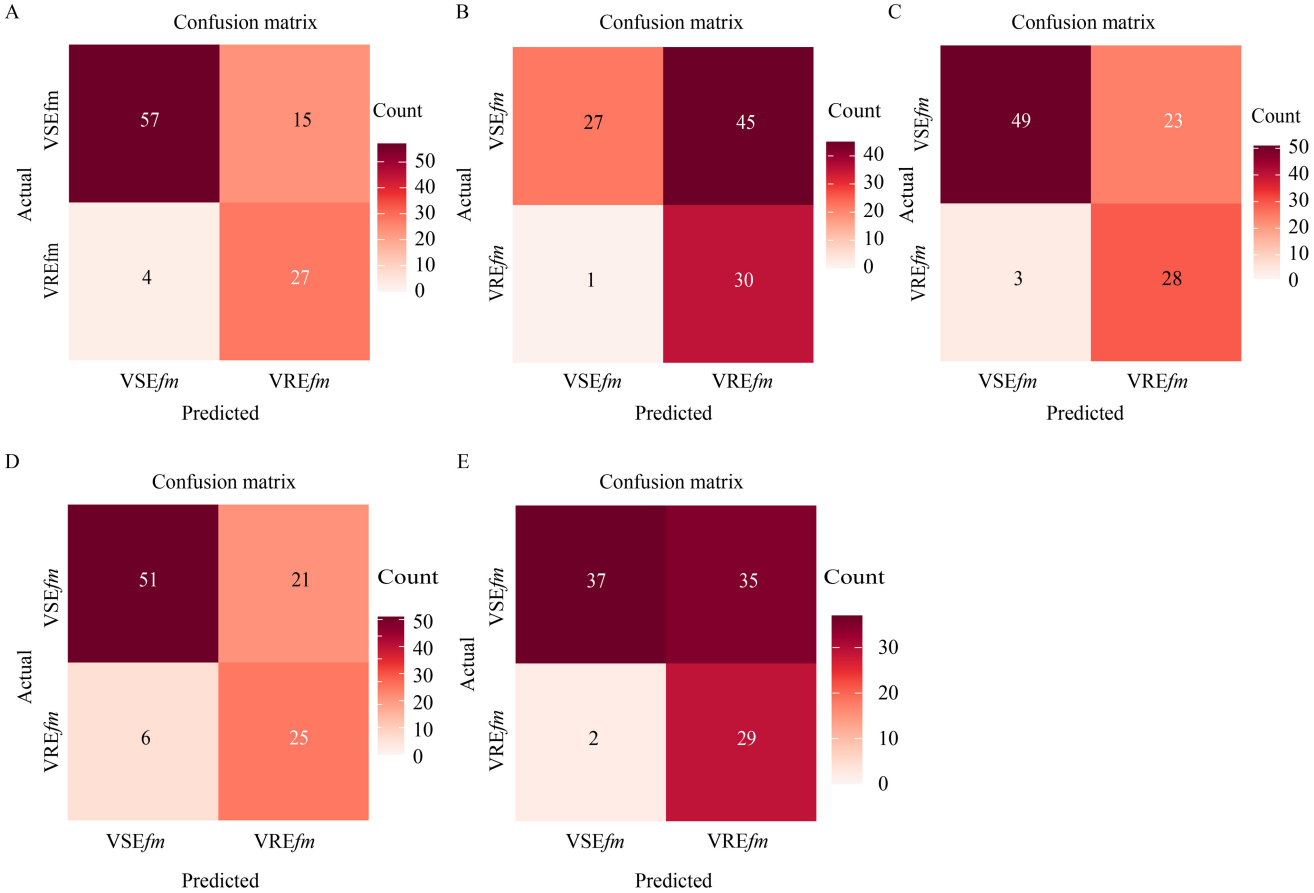
Confusion matrices of machine learning models in predicting VRE*fm* in the temporal external validation set. Panels **(A–E)** show the confusion matrices for the AdaBoostM1, BN, KNN, LR, and SVM models, respectively. Each matrix illustrates the classification results, with rows denoting actual classes (VSE*fm*: vancomycin-susceptible *Enterococcus faecium*; VRE*fm*: vancomycin-resistant *Enterococcus faecium*) and columns denoting predicted classes. The color gradient (from light to dark red) represents the number of samples in each class, with the color bar on the right showing the scale. AdaBoostM1, adaptive boosting algorithm M1; BN, Bayesian network; KNN, k-nearest neighbors; LR, logistic regression; SVM, support vector machine.

**Figure 6:**
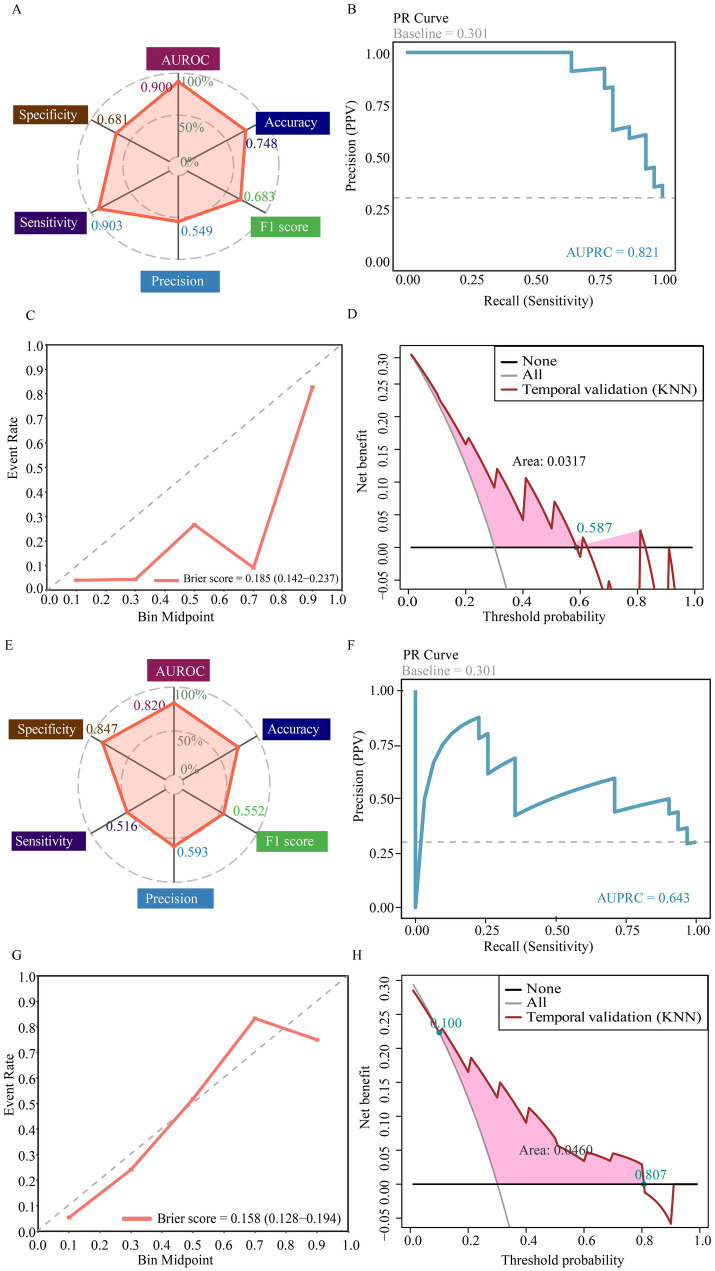
Comprehensive performance evaluation of the optimal KNN model on the temporal external validation set following 24-h **(A–D)** and 48-h **(E–H)** incubation. **(A,E)** Radar charts depicting six performance metrics. **(B,F)** Precision-recall curves with AUPRC indicated. **(C,G)** Calibration plots showing predicted probability vs. observed event rate. The black dashed line represents ideal calibration. **(D,H)** DCA comparing net benefit against treat-all and treat-none strategies; shaded areas denote integrated net benefit relative to treat-all. AUROC, area under the receiver operating characteristic curve; AUPRC, area under the precision-recall curve; DCA, decision curve analysis; KNN, k-nearest neighbors.

In the local cohort, Figures 3–6 summarized the classification performance of the five machine learning models. Figure 3 showed that, in the training set (5-fold cross-validation), all classifiers demonstrated strong discrimination (AUROC 0.953–0.986; AUPRC 0.960-0.981). Calibration curves and low Brier scores (range 0.04–0.06; Figure 4) indicated good concordance between predicted probabilities and observed outcomes. Similarly, Supplementary Table 3 showed consistently high *AB*_score_ values for all models (all >0.95). DCA confirmed a positive net clinical benefit across a range of threshold probabilities. Notably, LR classification exhibited pronounced departure from the ideal calibration line, indicating suboptimal probability calibration, whereas the other algorithms retained satisfactory calibration performance. Figure 4 further illustrated uniformly high performance across all models, with F1 scores consistently exceeding 0.90.

To further assess temporal generalizability, we validated the models using temporal validation isolates collected in 2025. Supplementary Figure 2 presents comprehensive performance metrics across eight evaluation measures, with KNN and AdaBoostM1 achieving the highest F1 scores (0.683 and 0.740, respectively). Figure 2B; Supplementary Table 4 further show that these two algorithms also attained the highest *AB*_score_ values (0.84 and 0.86, respectively). Based on calibration curves (Supplementary Figure 3A), KNN was selected as the optimal model. Performance on 24-h incubation data is shown in Figures 6A–D, achieving an AUROC of 0.900 and an accuracy of 0.748. Consistent with the AUROC, the AUPRC remained high at 0.821. Calibration analysis indicated acceptable overall calibration (Brier score, 0.185), with notable underestimation at lower predicted probabilities (<0.3) and progressively improved alignment as predicted probabilities increased, approaching the ideal line within the 0.7–0.9 range. DCA indicated that the KNN model offered incremental net clinical benefit over the treat-all approach across threshold probabilities from approximately 0 to 0.6. The standardized net benefit area was 0.0317.

To evaluate robustness under extended incubation conditions, the static KNN model (trained on 24-h spectra from 2020 to 2024) was applied to predict the 48-h mass spectra of the same temporal validation isolates (Figures 6E–H). The fixed KNN model maintained an AUROC of 0.820 but showed a marked decrease in sensitivity, dropping to 0.516, indicating an elevation in false-negative rates with prolonged culture.

### 3.4 Model interpretation and key feature analysis using Shapley values

**Figure 7:**
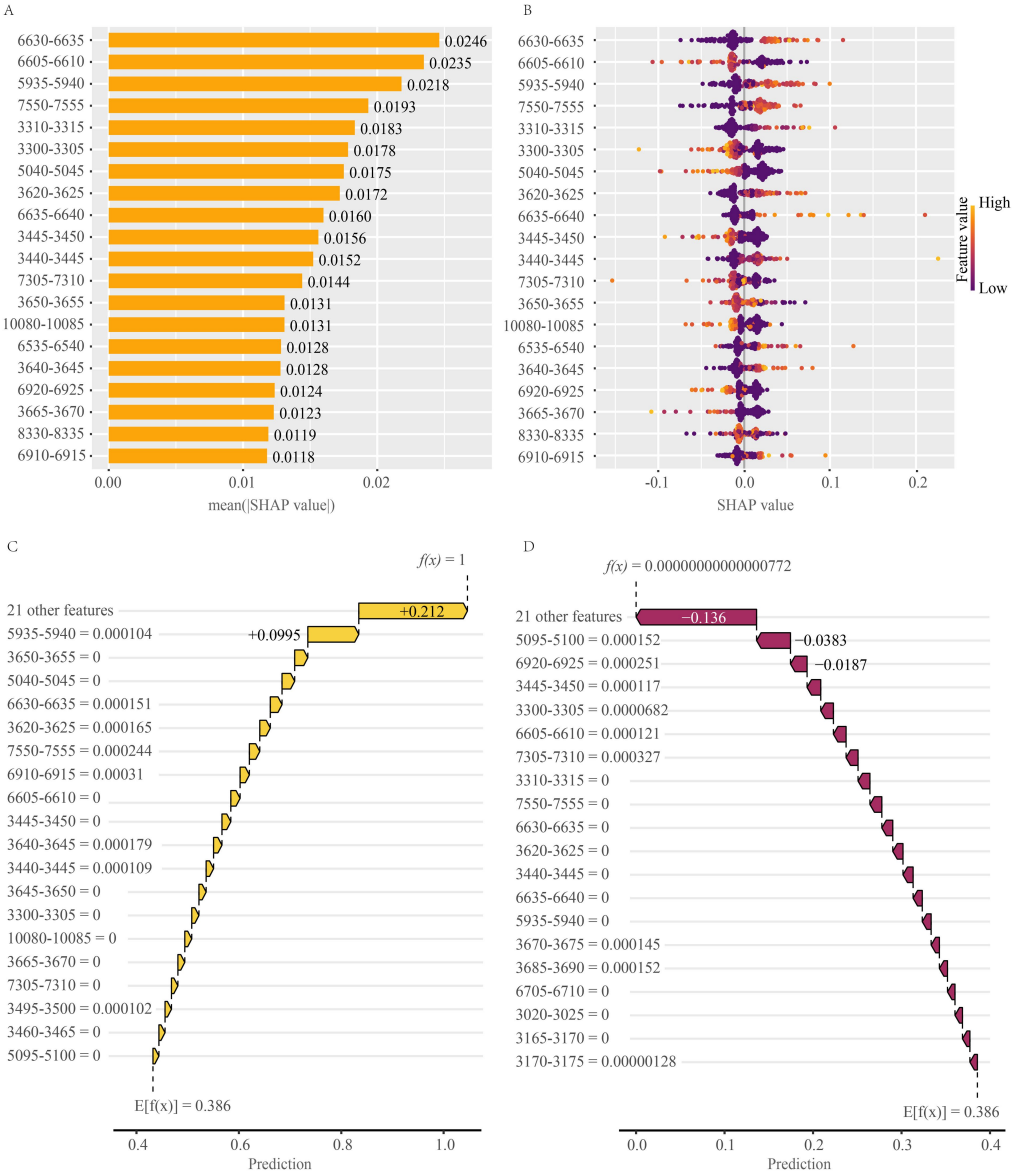
SHAP interpretability analysis of the optimal k-nearest neighbors (KNN) classifier for vancomycin resistance prediction in *Enterococcus faecium*. **(A)** Bar plot ranking the top 20 mass-to-charge ratio (*m/z*) features by mean absolute SHAP value. **(B)** Beeswarm plot illustrating the distribution and directionality of SHAP values for the top 20 features across all predictions; color intensity denotes feature magnitude (yellow, high; purple, low). **(C)** Waterfall plot for a representative vancomycin-resistant isolate (predicted probability = 1), showing feature contributions driving the prediction above the base value (*E*[*f*(*x*)] = 0.386). **(D)** Waterfall plot for a representative vancomycin-susceptible isolate (predicted probability ≈ 0), showing feature contributions driving the prediction below the base value. KNN, k-nearest neighbors; SHAP, SHapley Additive exPlanations.

To further evaluate the contribution of each feature of the best model (KNN), we calculated SHAP values at both the dataset and sample levels. Originating from cooperative game theory, SHAP values quantified each feature’s contribution to model outputs. Unlike Weis et al. (2022) (as detailed in Supplementary Table 5), who applied SHAP analysis on the entire feature set to select the top 30 influential variables, our study first employed XGBoost-RFE to identify a compact set of 40 discriminative *m/z* features. These features were subsequently ranked according to their mean absolute SHAP values in the final KNN model (Figure 7A). Notably, the top 12 *m/z* bins contributed substantially more than the remaining features. The SHAP summary plot (Figure 7B) depicts the relationship between feature value and SHAP contribution: where the color gradient represents feature magnitude, indicating that the predictor relies on either high-intensity values (yellow) or the absence of detected intensity (purple) to drive predictions for the positive (resistant) class. Specifically, for *m/z* bins 6,630–6,635, 5,935–5,940, 7,550–7,555, and 3,310–3,315, the presence of peaks is associated with the positive (resistant) class, whereas for *m/z* bin 6,605–6,610, the absence of peaks drives positive class prediction. We further observed that the majority of contributing features were located in the lower *m/z* range (<10,000 Da)., where more ions are typically measured in MALDI-TOF MS. SHAP waterfall plots were used to interpret individual predictions (Figures 7C,D): Figure 7C shows a vancomycin-resistant isolate correctly predicted with high resistance probability [*f*(*x*) = 1], mainly driven by the negative contribution of the *m/z* 5,935–5,940 bin (SHAP = +0.0995) combined with 21 other aggregated features; Figure 7D shows a sensitive isolate predicted with high confidence [*f*(*x*) ≈ 0], where *m/z* bins 5,095–5,100 (–0.0383), 6,920–6,925, and 3,445–3,450 made key negative contributions.

### 3.5 Multi-center data integration for local prediction enhancement

To evaluate cross-center generalizability, the optimal KNN model was externally validated on the independent DRIAMS-A dataset, achieving an AUROC of 0.536, indicating limited transportability across geographically distinct settings (Supplementary Figure 3B).

Drawing on prior evidence that simple data aggregation can outperform complex domain adaptation algorithms in clinical prediction tasks (Zhang et al., 2021), we subsequently assessed whether integrating multi-center data could enhance local predictive performance. As summarized in Table 1, KNN classifiers trained exclusively on external data—either DRIAMS-A alone or pooled DRIAMS subsets (A–D)—exhibited near-random discrimination when evaluated on the local 2025 temporal holdout set (AUROC 0.500 and 0.509, respectively). By contrast, hybrid configurations combining local training data with DRIAMS-A or pooled DRIAMS subsets markedly restored discriminative performance, achieving AUROCs of 0.826 and 0.818, respectively, with corresponding improvements in calibration (Brier score 0.122 and 0.167) and F1 score (0.733 and 0.687).

**Table 1:** Performance metrics for various training configurations.

Training cohort	AUROC	AUPRC	Brier score	F1 score
DRIAMS-A	0.500	0.301	0.301	–
DRIAMS-A + local	0.826	0.759	0.122	0.733
DRIAMS-A/B/C/D	0.509	0.278	0.294	–
DRIAMS-A/B/C/D + local	0.818	0.664	0.167	0.687

All models were evaluated using the local 2025 temporal holdout set. The local training set consisted of isolates collected during 2020–2024. AUROC, area under the receiver operating characteristic curve; AUPRC, area under the precision-recall curve; —, F1 score not applicable.

## 4 Discussion

### 4.1 Main findings of the study

In this study, machine learning classifiers trained on MALDI-TOF MS spectral data demonstrated robust predictive performance for vancomycin resistance in *E. faecium* across both temporal and spatial validation frameworks. Notably, hybrid local-external configurations maintained clinically acceptable performance during spatial validation, suggesting that augmenting limited local datasets with external spectral repositories can overcome sample scarcity without compromising discriminative accuracy. This strategy offers a pragmatic solution for laboratories seeking to implement MALDI-TOF MS–based resistance prediction in the absence of established local surveillance infrastructure.

### 4.2 Model selection and comparison with deep learning methods

This study selected five algorithms, AdaBoostM1, BN, KNN, LR, and SVM, to construct a VRE*fm* prediction model. This selection aligns closely with established algorithmic frameworks in the field of MALDI-TOF MS resistance prediction. Existing studies have confirmed that LR, SVM, KNN, Random Forest (RF), and Light Gradient Boosting Machine (LightGBM) are classic algorithms in this field (Wang et al., 2021; Weis et al., 2022; Gao et al., 2024; Wiesmann et al., 2025). Building on this foundation, the present study incorporated ensemble learning models, such as AdaBoostM1, to further validate the adaptability of nonlinear models to high-dimensional spectral data.

Although deep learning models such as MLP have been included in the analysis of multiple related studies (Weis et al., 2022; Gao et al., 2024; Wiesmann et al., 2025), this study did not prioritize the use of deep models. The primary rationale for this decision stems from the study’s design as a single-center investigation with a limited sample size (*n* = 268). Deep learning models require large-scale annotated data to avoid overfitting, and their “black box” nature leads to insufficient clinical interpretability, making it difficult to meet the clinical need for tracing prediction mechanisms. Additionally, deep models have high hardware and process modification thresholds, which do not align with this study’s goal of pursuing rapid and convenient clinical translation. The nonlinear models employed in this study demonstrated excellent generalization ability following feature selection, achieved interpretability via SHAP analysis, exhibited low computational costs, and allowed for seamless integration into existing mass spectrometry workflows. Subsequent research will expand multi-center samples, construct deep learning models such as MLP, compare and verify their performance with the optimal model from this study, and further improve the prediction system.

### 4.3 Variability of characteristic peaks and underlying causes

In this study, the top five characteristic peaks (*m/z* bins 6,630–6,635, 6,605–6,610, 5,935–5,940, 7,550–7,555, and 3,310–3,315) were identified as key discriminators between VRE*fm* and VSE*fm*. Nevertheless, previous research has reported varying results regarding the characteristic peaks used for this distinction. For instance, specific ions at *m/z* 3,184, 5,702, 7,415, 7,445, and 12,662 have been identified as distinguishing peaks (Nakano et al., 2014). Another study identified ions at m/z 2,211.5, 5,094.7, 5,945.7, and 8,327.9 as characteristic peaks (Griffin et al., 2012). Additionally, ions at *m/z* 3,516.14 and 3,644.12 were reported as characteristic peaks for this distinction (Wang et al., 2014). This inconsistency may be due to various reasons. First, the prevalence of VRE*fm* varies in different countries and regions, and the composition of isolates may also differ across areas. Therefore, compared to general models, machine learning models trained on local data tend to provide better performance. Second, different sample processing methods may affect the number of peaks. Third, different methods of selecting primary peaks may contribute to the inconsistent results. Previous research has primarily used linear methods for feature selection, including L1-SVM, chi-square tests, and traditional statistical analyses (Griffin et al., 2012; Wang et al., 2014), which makes it difficult to capture nonlinear interactions between mass spectrometry peaks. The XGBoost-RFE algorithm introduced in this study was able to identify complex associations between features by employing ensemble learning strategies. This approach efficiently screened out the 40 *m/z* ranges with the highest cumulative contribution rate, from a small sample cohort of 268 strains (Figure 2A). These factors collectively contribute to the observed distinct discriminating peaks.

### 4.4 Clinical utility despite imperfect sensitivity

The KNN classifier achieved an AUROC of 0.820 in temporal validation, but its sensitivity was 0.516, meaning approximately 48.4% of true VRE*fm* cases would be misclassified as susceptible. This misclassification rate needs careful consideration in the anticipated clinical application scenario. It must be emphasized that the proposed MALDI-TOF MS-based machine learning method is not intended to replace phenotypic AST, but rather to serve as a rapid screening triage tool, with its core value lying in compressing the drug resistance alert window. While traditional AST typically requires 48–72 h, MALDI-TOF MS can complete species identification within 24 h of obtaining the strain, simultaneously providing a drug resistance risk signal. Therefore, the envisioned clinical workflow is as follows: a predicted resistant result triggers immediate contact isolation and adjustment of empirical antibiotic therapy, whereas a predicted susceptible result is subjected directly to standard AST confirmation without additional delay. This screening-confirmation model strikes a balance between accelerating decision-making and the diagnostic accuracy of phenotypic testing.

For patient safety and infection control, the key metric is not sensitivity alone, but the net reduction in unprotected exposure days. Even with a sensitivity of 51.6%, early identification of most VRE*fm* cases compared to the conventional AST workflow could shorten the time window for uncontrolled nosocomial transmission by 24–48 h through immediate contact precautions. DCA supports this interpretation: the model demonstrated net benefit superior to the “treat all” strategy across a threshold probability range from 0.100 to 0.807 (Figure 6H), indicating clinical utility despite imperfect discrimination. However, the 48.4% false negative rate poses a tangible risk: missed VRE*fm* cases may receive inappropriate vancomycin treatment without isolation, potentially exacerbating outbreak clusters in high-prevalence departments. To address this safety gap, we propose a risk-stratified point-of-care testing protocol: predicted susceptible strains from the ICU, hematology-oncology department, or those from patients with prior VRE colonization will undergo accelerated AST testing, while isolates from low-risk wards will follow standard turnaround times. This stratified approach balances the speed advantage of MALDI-TOF MS prediction with the safety cost of residual false negatives.

### 4.5 Comparison with PCR-based resistance detection

Although our MALDI-TOF MS workflow reduces turnaround time compared with phenotypic AST (Supplementary Figure 1), its role compared to PCR-based methods warrants further investigation. Automated PCR platforms such as the Cepheid GeneXpert vanA/vanB assay deliver results in under 1 h, though typically requiring approximately 2 h from rectal swabs, with high sensitivity for known vanA/vanB determinants (Keidar-Friedman et al., 2025). The BioFire FilmArray BCID2 detects pathogens and resistance genes from positive blood cultures within approximately 3.5 h (Caméléna et al., 2023). However, these require dedicated infrastructure, trained molecular personnel, and are restricted to predefined genetic targets. By contrast, our framework leverages existing infrastructure for routine species identification. Once a pure colony is available—within 18–24 h of primary culture—spectral acquisition takes seconds to minutes per isolate, facilitating high-throughput batch processing via the MALDI-TOF platform (Duncan and DeMarco, 2019). With accelerated incubation protocols, the total time from blood culture positivity to prediction can be reduced to 4 h while requiring no additional capital expenditure or specialized expertise (Kohlmann et al., 2015).

Beyond turnaround time considerations, the critical distinction lies in the biological target. PCR detects specific genetic determinants and may miss novel or variant resistance mechanisms, such as vanM found in *Enterococcus faecium* (Xu et al., 2010). Given our study did not genotype isolates, a PCR-negative result would yield false reassurance for resistance mediated by alternative mechanisms. Our spectral approach captures proteomic signatures associated with the resistant phenotype and remains agnostic to genetic backgrounds. This phenotypic prediction paradigm is supported by our cross-center validation results (Table 1). Cost structures differ substantially. PCR assays incur per-test cartridge costs and depend on continuous reagent supply chains, whereas MALDI-TOF MS prediction, once trained, utilizes standard matrix and target plates already stocked for species identification. In resource-limited laboratories or settings where VRE prevalence is sporadic—where PCR infrastructure costs cannot be effectively amortized—this zero-additional-infrastructure approach facilitates pragmatic deployment. Nevertheless, PCR retains value for culture-negative specimens and non-viable organisms (Adzitey et al., 2013; Galhano et al., 2021), scenarios beyond culture-dependent MALDI-TOF MS workflows.

### 4.6 Generalizability constraints and underlying biases

To evaluate the generalizability of the model, we conducted temporal validation using an independent dataset collected in 2025. The results showed that the model’s performance on the temporal validation set declined compared to that on the training set. This discrepancy likely stems primarily from data leakage and associated spectral similarity bias, driven chiefly by clonal bias. Since this study did not employ multilocus sequence typing (MLST) or whole-genome sequencing (WGS), we could not definitively exclude the presence of closely related dominant hospital clones in the training data at the molecular level. If such clone isolates co-occurred in the training set, their high genetic similarity would lead to highly similar MALDI-TOF MS spectra. Consequently, when such highly similar samples were present in both the randomly partitioned training and validation sets, the evaluation of model performance might be overestimated. Therefore, the model may be more adept at identifying specific clones rather than general antimicrobial resistance genes, thereby weakening its predictive ability for newly emerging external clones. As shown in Supplementary Figure 3B; Table 1, external-only models performed near chance level (AUROC 0.500–0.536), in stark contrast to the clinically acceptable performance of hybrid local-external strategies (AUROC > 0.80), provides indirect support for the hypothesis of substantial domain shift across geographically distinct bacterial populations. This suggests that while multi-center repositories broaden lineage coverage, they remain insufficient for transferable AMR prediction without adequate local calibration to align the model with institution-specific clonal structures. Notably, several characteristic peaks identified in this study correspond to biomarkers previously reported across different continents (Griffin et al., 2012) (e.g., *m/z* 5,095; Supplementary Table 2), suggesting that these features may exhibit cross-clonal consistency. Furthermore, performance fluctuations may also be related to the inherent temporal variations in antimicrobial resistance prevalence. This implies differences in the class ratios of resistant versus sensitive strains between the training set and the external validation set.

### 4.7 Identification of characteristic MS peaks linked to VRE*fm* drug resistance

The clinical relevance of VRE*fm* depends on clarifying its resistance mechanisms, and one promising strategy is to identify reproducible mass spectrometry peaks across independent studies. In the present analysis we detected predictive peaks within the *m/z* 3,640–3,645 interval (Supplementary Table 2), including *m/z* 3,644.12, which has been strongly linked to the vanA genotype (Wang et al., 2014). Importantly, an independent investigation resolved the chemical identity of a key peak in this region (*m/z* 3,645) by protein purification and tandem mass spectrometry, demonstrating that it corresponds to a doubly charged, acetylated form of the 30S ribosomal protein S14 Z-type (RS14Z_ENTFA). That modified protein was more prevalent in VRE*fm* than in VSE*fm* (Wang et al., 2021), suggesting a possible novel mechanism contributing to resistance.

Other groups have likewise reported distinctive peaks with molecular correlates. A stable peak at *m/z* 5,094.7 in vanB-type VRE*fm* was originally identified (Griffin et al., 2012), later attributed to the bacteriocin hiracin encoded by *hirJM79* (Brackmann et al., 2020). Gao et al. (2024)identified a peak at *m/z* ~ 7,328 as the ATP synthase c subunit (A0A133N352), which may influence bacterial survival under antibiotic stress, and a peak at *m/z* ~ 7,344 as the large ribosomal subunit protein uL29 (R2Q455). Because uL29 is a known antibiotic target (Auerbach et al., 2002), its association provides direct mechanistic rationale for its use as a resistance biomarker.

These consistently observed spectral features across studies not only enhance the reliability of peak-based classification but also highlight candidate molecular targets worthy of in-depth investigation. Elucidating the biological associations of these characteristic peaks will not only help validate the biological basis of the models but may ultimately reveal new therapeutic avenues for combating VRE*fm* infections.

### 4.8 Limitations

This study has several limitations. Firstly, vanA/vanB genotypes were not confirmed by PCR or WGS, so the model yields only phenotypic predictions and cannot attribute resistance to specific molecular mechanisms. Secondly, several characteristic mass-spectrometry peaks remain unassigned to known resistance pathways, and their biological relevance requires experimental clarification. Thirdly, although we assessed robustness to extended 48-h incubation, our study did not systematically investigate different culture media, temperatures, or additional pre-analytical variables. Finally, prospective validation under standardized protocols is needed to demonstrate real-time performance and clinical utility; subsequent work should also evaluate integration of the model into clinical decision-support tools and laboratory information systems for routine use.

## 5 Conclusion

Overall, this study demonstrates that machine learning analysis of MALDI-TOF MS spectral data provides a novel approach for predicting vancomycin resistance in *E. faecium*, achieving robust discriminative performance across both local and multi-center enhanced training configurations. These findings support a resource-efficient implementation strategy for clinical microbiology laboratories through the active integration of public spectral repositories to mitigate local sample constraints. This hybrid approach may accelerate the clinical translation of MALDI-TOF MS–based resistance prediction into routine practice.

## Data Availability

The original contributions presented in the study are included in the article/Supplementary material, further inquiries can be directed to the corresponding author.

## Footnotes

1
https://doi.org/10.5061/dryad.bzkh1899q

## Supplementary material

Supplementary Figure 1 Comparison of turnaround times from sample receipt to species identification by MALDI-TOF MS and phenotypic resistance testing by conventional antimicrobial susceptibility testing (AST). Turnaround times were measured for patient samples collected in 2025 at the local diagnostic laboratory. Box plots display the median (horizontal line), interquartile range (box boundaries, 25th and 75th percentiles), and adjacent values (whiskers).

Supplementary Figure 2 Heatmap of machine learning model performance metrics on the temporal external validation set. The heatmap displays the performance of five models (AdaBoostM1, BN, KNN, LR, SVM) across eight key evaluation metrics. The color gradient (from blue to red) represents the magnitude of the metric values, with the color bar on the right denoting the scale. AdaBoostM1, adaptive boosting algorithm M1; BN, Bayesian network; KNN, k-nearest neighbors; LR, logistic regression; SVM, support vector machine.

Supplementary Figure 3 Model calibration and spatial external validation. **(A)** Calibration curves for five models (AdaBoostM1, BN, KNN, LR, SVM) on the temporal external validation set. The black dashed line represents ideal calibration; shaded areas denote 95% CI. **(B)** ROC curve of the KNN classifier on the DRIAMS-A spatial external validation cohort. AdaBoostM1, adaptive boosting algorithm M1; BN, Bayesian network; KNN, k-nearest neighbors; LR, logistic regression; SVM, support vector machine; AUROC, area under the receiver operating characteristic curve; CI, confidence interval.

## References

[ref1] Adzitey F. Huda N. Ali G. R. (2013). Molecular techniques for detecting and typing of bacteria, advantages and application to foodborne pathogens isolated from ducks. 3 Biotech 3, 97–107. doi: 10.1007/s13205-012-0074-4, 28324565 PMC3597138

[ref2] Auerbach T. Bashan A. Harms J. Schluenzen F. Zarivach R. Bartels H. . (2002). Antibiotics targeting ribosomes: crystallographic studies. Curr. Drug Targets Infect. Disord. 2, 169–186. doi: 10.2174/1568005023342506 12462147

[ref3] Bifarin O. O. (2023). Interpretable machine learning with tree-based shapley additive explanations: application to metabolomics datasets for binary classification. PLoS One 18:e0284315. doi: 10.1371/journal.pone.0284315, 37141218 PMC10159207

[ref4] Bobenchik A. M. Deak E. Hindler J. A. Charlton C. L. Humphries R. M. (2015). Performance of Vitek 2 for antimicrobial susceptibility testing of Enterobacteriaceae with Vitek 2 (2009 FDA) and 2014 CLSI breakpoints. J. Clin. Microbiol. 53, 816–823. doi: 10.1128/jcm.02697-14, 25540403 PMC4390649

[ref5] Brackmann M. Leib S. L. Tonolla M. Schürch N. Wittwer M. (2020). Antimicrobial resistance classification using MALDI-TOF-MS is not that easy: lessons from vancomycin-resistant *Enterococcus faecium*. Clin. Microbiol. Infect. 26, 391–393. doi: 10.1016/j.cmi.2019.10.027, 31682986

[ref6] Caméléna F. Péan de Ponfilly G. Pailhoriès H. Bonzon L. Alanio A. Poncin T. . (2023). Multicenter evaluation of the FilmArray blood culture identification 2 panel for pathogen detection in bloodstream infections. Microbiol. Spectrum 11:e0254722. doi: 10.1128/spectrum.02547-22, 36519852 PMC9927563

[ref7] Chen J. H. K. Cheng V. C. C. Wong C. P. Wong S. C. Y. Yam W. C. Yuen K. Y. (2017). Rapid differentiation of *Haemophilus influenzae* and *Haemophilus haemolyticus* by use of matrix-assisted laser desorption ionization-time of flight mass spectrometry with ClinProTools mass spectrum analysis. J. Clin. Microbiol. 55, 2679–2685. doi: 10.1128/jcm.00267-17, 28637909 PMC5648705

[ref8] Cho Y. R. Kang M. (2020). Interpretable machine learning in bioinformatics. Methods 179, 1–2. doi: 10.1016/j.ymeth.2020.05.024, 32479800

[ref9] Clinical and Laboratory Standards Institute (2019). Performance Standards for Antimicrobial Susceptibility Testing; M100. 29th Edn Wayne: Clinical and Laboratory Standards Institute.

[ref10] Collins G. S. Moons K. G. M. Dhiman P. Riley R. D. Beam A. L. Van Calster B. . (2024). TRIPOD+AI statement: updated guidance for reporting clinical prediction models that use regression or machine learning methods. BMJ 385:e078378. doi: 10.1136/bmj-2023-078378.10.1136/bmj-2023-, 38626948 PMC11019967

[ref11] da Silva N. S. Muniz V. D. Estofolete C. F. Furtado G. H. Rubio F. G. (2014). Identification of temporal clusters and risk factors of bacteremia by nosocomial vancomycin-resistant *Enterococci*. Am. J. Infect. Control 42, 389–392. doi: 10.1016/j.ajic.2013.11.010, 24679566

[ref12] Duncan M. DeMarco M. L. (2019). MALDI-MS: emerging roles in pathology and laboratory medicine. Clin. Mass Spectrom. 13, 1–4. doi: 10.1016/j.clinms.2019.05.003, 34841079 PMC8620520

[ref13] EUCAST. (2019). The European Committee on Antimicrobial Susceptibility Testing. Breakpoint tables for interpretation of MICs and zone diameters. Version 9.0. Available online at: http://www.eucast.org.

[ref14] Fiore E. Van Tyne D. Gilmore M. S. (2019). Pathogenicity of enterococci. Microbiol. Spectr. 7:4. doi: 10.1128/microbiolspec.GPP3-0053-2018 PMC6629438 31298205

[ref15] Forrest G. N. Roghmann M. C. Toombs L. S. Johnson J. K. Weekes E. Lincalis D. P. . (2008). Peptide nucleic acid fluorescent in situ hybridization for hospital-acquired enterococcal bacteremia: delivering earlier effective antimicrobial therapy. Antimicrob. Agents Chemother. 52, 3558–3563. doi: 10.1128/aac.00283-08, 18663022 PMC2565911

[ref16] Gajic I. Kabic J. Kekic D. Jovicevic M. Milenkovic M. Mitic Culafic D. . (2022). Antimicrobial susceptibility testing: a comprehensive review of currently used methods. Antibiotics (Basel) 11:427. doi: 10.3390/antibiotics11040427, 35453179 PMC9024665

[ref17] Galhano B. S. P. Ferrari R. G. Panzenhagen P. de Jesus A. C. S. Conte-Junior C. A. (2021). Antimicrobial resistance gene detection methods for bacteria in animal-based foods: a brief review of highlights and advantages. Microorganisms 9:923. doi: 10.3390/microorganisms9050923, 33925810 PMC8146338

[ref18] Gao W. Li H. Yang J. Zhang J. Fu R. Peng J. . (2024). Machine learning assisted MALDI mass spectrometry for rapid antimicrobial resistance prediction in clinicals. Anal. Chem. 96, 13398–13409. doi: 10.1021/acs.analchem.4c00741, 39096240

[ref19] García-Solache M. Rice L. B. (2019). The Enterococcus: a model of adaptability to its environment. Clin. Microbiol. Rev. 32:2. doi: 10.1128/cmr.00058-18, 30700430 PMC6431128

[ref20] Gibb S. Strimmer K. (2012). MALDIquant: a versatile R package for the analysis of mass spectrometry data. Bioinformatics 28, 2270–2271. doi: 10.1093/bioinformatics/bts447, 22796955

[ref21] Gök Ş. M. Türk Dağı H. Kara F. Arslan U. Fındık D. (2020). Investigation of antibiotic resistance and virulence factors of *Enterococcus faecium* and *Enterococcus faecalis* strains isolated from clinical samples. Mikrobiyol. Bul. 54, 26–39. doi: 10.5578/mb.68810, 32050876

[ref22] Griffin P. M. Price G. R. Schooneveldt J. M. Schlebusch S. Tilse M. H. Urbanski T. . (2012). Use of matrix-assisted laser desorption ionization-time of flight mass spectrometry to identify vancomycin-resistant enterococci and investigate the epidemiology of an outbreak. J. Clin. Microbiol. 50, 2918–2931. doi: 10.1128/jcm.01000-12, 22740710 PMC3421795

[ref23] Hegstad K. Giske C. G. Haldorsen B. Matuschek E. Schønning K. Leegaard T. M. . (2014). Performance of the EUCAST disk diffusion method, the CLSI agar screen method, and the Vitek 2 automated antimicrobial susceptibility testing system for detection of clinical isolates of enterococci with low- and medium-level VanB-type vancomycin resistance: a multicenter study. J. Clin. Microbiol. 52, 1582–1589. doi: 10.1128/jcm.03544-13, 24599985 PMC3993630

[ref24] Hrabák J. Chudácková E. Walková R. (2013). Matrix-assisted laser desorption ionization-time of flight (MALDI-TOF) mass spectrometry for detection of antibiotic resistance mechanisms: from research to routine diagnosis. Clin. Microbiol. Rev. 26, 103–114. doi: 10.1128/cmr.00058-12, 23297261 PMC3553667

[ref25] Hughes H. Y. Odom R. T. Michelin A. V. Snitkin E. S. Sinaii N. Milstone A. M. . (2019). A retrospective cohort study of antibiotic exposure and vancomycin-resistant *Enterococcus* recolonization. Infect. Control Hosp. Epidemiol. 40, 414–419. doi: 10.1017/ice.2019.15, 30729903

[ref26] Keidar-Friedman D. Gil L. Tsur A. Brosh-Nissimov T. Carmeli Y. Rosenfeld B. D. . (2025). Evaluation of sample pooling using Xpert Carba-R and Xpert vanA/vanB PCR for screening of carbapenemase-producing Enterobacterales and vancomycin-resistant Enterococcus colonization. Microbiol. Spectrum 13:e0108025. doi: 10.1128/spectrum.01080-25, 40899812 PMC12502523

[ref27] Kim J. M. Kim I. Chung S. H. Chung Y. Han M. Kim J. S. (2019). Rapid discrimination of methicillin-resistant *Staphylococcus aureus* by MALDI-TOF MS. Pathogens 8:214. doi: 10.3390/pathogens8040214, 31683799 PMC6963962

[ref28] Kohlmann R. Hoffmann A. Geis G. Gatermann S. (2015). MALDI-TOF mass spectrometry following short incubation on a solid medium is a valuable tool for rapid pathogen identification from positive blood cultures. Int. J. Med. Microbiol. 305, 469–479. doi: 10.1016/j.ijmm.2015.04.004, 25953498

[ref29] Li W. Wang B. Li T. Ma Y. Jin H. Zhao J. . (2026). A causal and interpretable machine learning framework for postcranioplasty risk prediction and surgical decision support. NPJ Digit. Med. 9:1. doi: 10.1038/s41746-026-02370-6, 41566002 PMC12923646

[ref30] López-Cortés X. A. Manríquez-Troncoso J. M. Sepúlveda A. Y. Soto P. S. (2025). Integrating machine learning with MALDI-TOF mass spectrometry for rapid and accurate antimicrobial resistance detection in clinical pathogens. Int. J. Mol. Sci. 26:1140. doi: 10.3390/ijms26031140, 39940908 PMC11817502

[ref31] Mather C. A. Werth B. J. Sivagnanam S. SenGupta D. J. Butler-Wu S. M. (2016). Rapid detection of vancomycin-intermediate *Staphylococcus aureus* by matrix-assisted laser desorption ionization-time of flight mass spectrometry. J. Clin. Microbiol. 54, 883–890. doi: 10.1128/jcm.02428-15, 26763961 PMC4809916

[ref32] Nakano S. Matsumura Y. Kato K. Yunoki T. Hotta G. Noguchi T. . (2014). Differentiation of vanA-positive *Enterococcus faecium* from vanA-negative *E. faecium* by matrix-assisted laser desorption/ionisation time-of-flight mass spectrometry. Int. J. Antimicrob. Agents 44, 256–259. doi: 10.1016/j.ijantimicag.2014.05.006, 25104134

[ref33] Pizzato J. Tang W. Bernabeu S. Bonnin R. A. Bille E. Farfour E. . (2022). Discrimination of *Escherichia coli*, *Shigella flexneri*, and *Shigella sonnei* using lipid profiling by MALDI-TOF mass spectrometry paired with machine learning. Microbiologyopen 11:e1313. doi: 10.1002/mbo3.1313, 36004556 PMC9405496

[ref34] Poirion O. B. Jing Z. Chaudhary K. Huang S. Garmire L. X. (2021). DeepProg: an ensemble of deep-learning and machine-learning models for prognosis prediction using multi-omics data. Genome Med. 13:112. doi: 10.1186/s13073-021-00930-x, 34261540 PMC8281595

[ref35] Reynolds J. L. Trudeau R. E. Seville M. T. Chan L. (2021). Impact of a vancomycin-resistant *Enterococcus* (VRE) screening result on appropriateness of antibiotic therapy. Antimicrob. Steward. Healthc. Epidemiol. 1:e41. doi: 10.1017/ash.2021.215, 36168474 PMC9495624

[ref36] Sati H. Carrara E. Savoldi A. Hansen P. Garlasco J. Campagnaro E. . (2025). The WHO bacterial priority pathogens list 2024: a prioritisation study to guide research, development, and public health strategies against antimicrobial resistance. Lancet Infect. Dis. 25, 1033–1043. doi: 10.1016/s1473-3099(25)00118-5, 40245910 PMC12367593

[ref37] Somily A. M. Al-Mohizea M. M. Absar M. M. Fatani A. J. Ridha A. M. Al-Ahdal M. N. . (2016). Molecular epidemiology of vancomycin resistant enterococci in a tertiary care hospital in Saudi Arabia. Microb. Pathog. 97, 79–83. doi: 10.1016/j.micpath.2016.05.019, 27247096

[ref38] Stagliano D. R. Susi A. Adams D. J. Nylund C. M. (2021). Epidemiology and outcomes of vancomycin-resistant *Enterococcus* infections in the U.S. military health system. Mil. Med. 186, 100–107. doi: 10.1093/milmed/usaa229, 33499465

[ref39] Stevens R. J. Poppe K. K. (2020). Validation of clinical prediction models: what does the “calibration slope” really measure? J. Clin. Epidemiol. 118, 93–99. doi: 10.1016/j.jclinepi.2019.09.016, 31605731

[ref40] Top J. Willems R. Blok H. de Regt M. Jalink K. Troelstra A. . (2007). Ecological replacement of *Enterococcus faecalis* by multiresistant clonal complex 17 *Enterococcus faecium*. Clin. Microbiol. Infect. 13, 316–319. doi: 10.1111/j.1469-0691.2006.01631.x, 17391388

[ref41] Tripathi A. Shukla S. K. Singh A. Prasad K. N. (2016). Prevalence, outcome and risk factor associated with vancomycin-resistant *Enterococcus faecalis* and *Enterococcus faecium* at a tertiary care hospital in northern India. Indian J. Med. Microbiol. 34, 38–45. doi: 10.4103/0255-0857.174099, 26776117

[ref42] Vickers A. J. Elkin E. B. (2006). Decision curve analysis: a novel method for evaluating prediction models. Med. Decis. Mak. 26, 565–574. doi: 10.1177/0272989x06295361, 17099194 PMC2577036

[ref43] Wang H. Y. Chung C. R. Chen C. J. Lu K. P. Tseng Y. J. Chang T. H. . (2021). Clinically applicable system for rapidly predicting *Enterococcus faecium* susceptibility to vancomycin. Microbiol. Spectrum 9:e0091321. doi: 10.1128/Spectrum.00913-21, 34756065 PMC8579932

[ref44] Wang H. Y. Hsieh T. T. Chung C. R. Chang H. C. Horng J. T. Lu J. J. . (2022). Efficiently predicting vancomycin resistance of *Enterococcus faecium* from MALDI-TOF MS spectra using a deep learning-based approach. Front. Microbiol. 13:821233. doi: 10.3389/fmicb.2022.821233, 35756017 PMC9231590

[ref45] Wang L. J. Lu X. X. Wu W. Sui W. J. Zhang G. (2014). Application of matrix-assisted laser desorption ionization time-of-flight mass spectrometry in the screening of vanA-positive *Enterococcus faecium*. Eur. J. Mass Spectrom. (Chichester) 20, 461–465. doi: 10.1255/ejms.1298, 25905870

[ref46] Weis C. Cuénod A. Rieck B. Dubuis O. Graf S. Lang C. . (2022). Direct antimicrobial resistance prediction from clinical MALDI-TOF mass spectra using machine learning. Nat. Med. 28, 164–174. doi: 10.1038/s41591-021-01619-9, 35013613

[ref47] Wiesmann N. Enders D. Westendorf A. Koch R. Schaumburg F. (2025). Prediction of antimicrobial resistance from MALDI-TOF mass spectra using machine learning: a validation study. J. Clin. Microbiol. 63:e0118625. doi: 10.1128/jcm.01186-25, 41296602 PMC12710331

[ref48] Wisplinghoff H. Bischoff T. Tallent S. M. Seifert H. Wenzel R. P. Edmond M. B. (2004). Nosocomial bloodstream infections in US hospitals: analysis of 24,179 cases from a prospective nationwide surveillance study. Clin. Infect. Dis. 39, 309–317. doi: 10.1086/421946, 15306996

[ref49] Xiao D. Zhao F. Zhang H. Meng F. Zhang J. (2014). Novel strategy for typing *Mycoplasma pneumoniae* isolates by use of matrix-assisted laser desorption ionization-time of flight mass spectrometry coupled with ClinProTools. J. Clin. Microbiol. 52, 3038–3043. doi: 10.1128/jcm.01265-14, 24920781 PMC4136174

[ref50] Xu X. Lin D. Yan G. Ye X. Wu S. Guo Y. . (2010). vanM, a new glycopeptide resistance gene cluster found in *Enterococcus faecium*. Antimicrob. Agents Chemother. 54, 4643–4647. doi: 10.1128/aac.01710-09, 20733041 PMC2976141

[ref51] Zhang H. Dullerud N. Seyyed-Kalantari L. Morris Q. Joshi S. Ghassemi M. (2021). "An empirical framework for domain generalization in clinical settings," in Proceedings of the Conference on Health, Inference, and Learning (CHIL '21). (Virtual Event, USA: Association for Computing Machinery), 279–290.

