# Supplementary figures and images for: Rapid prediction of vancomycin-resistant Enterococcus faecium using MALDI-TOF mass spectrometry and machine learning
Source: Front Microbiol. 2026 Jun 16;17:1789841. doi: 10.3389/fmicb.2026.1789841 (PMC13314634; doi:10.3389/fmicb.2026.1789841)

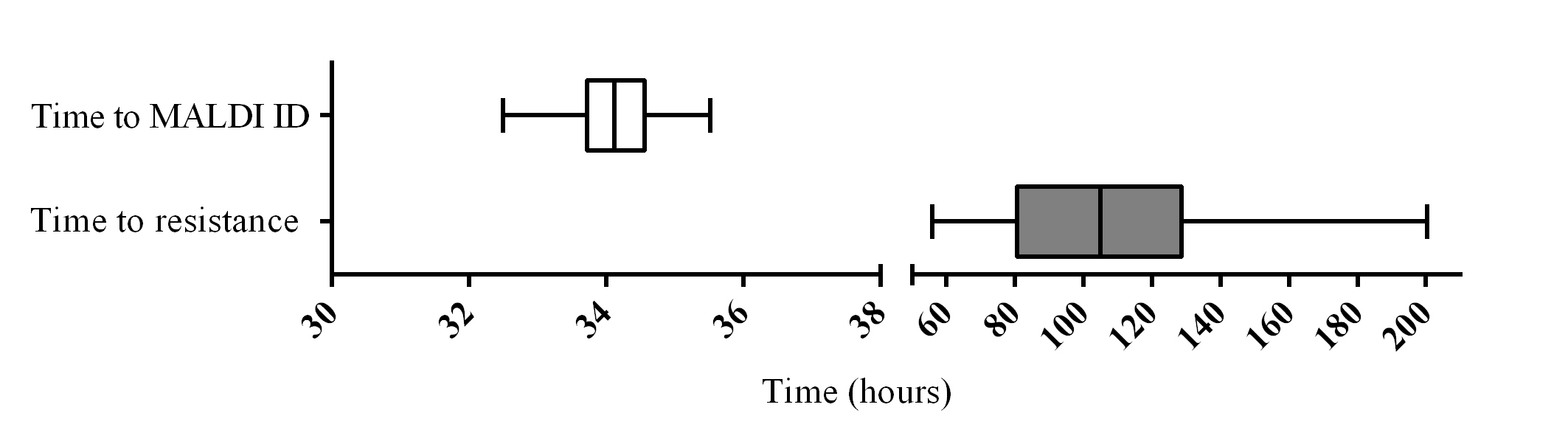

Supplement: Supplementary Figure 1 — Comparison of turnaround times from sample receipt to species identification by MALDI-TOF MS and phenotypic resistance testing by conventional antimicrobial susceptibility testing (AST). Turnaround times were measured for patient samples collected in 2025 at the local diagnostic laboratory. Box plots display the median (horizontal line), interquartile range (box boundaries, 25th and 75th percentiles), and adjacent values (whiskers). [file Image_1.jpg]

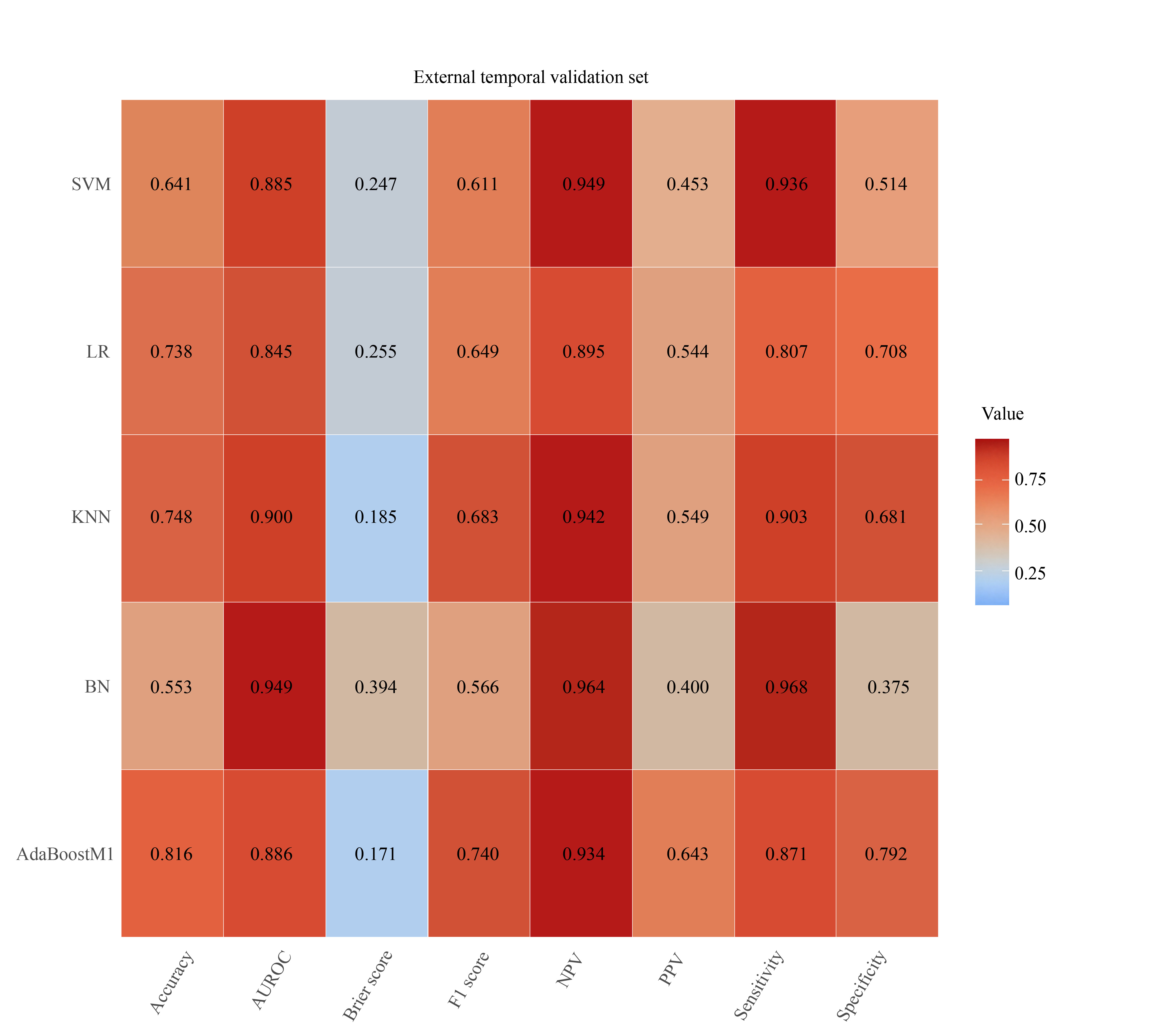

Supplement: Supplementary Figure 2 — Heatmap of machine learning model performance metrics on the temporal external validation set. The heatmap displays the performance of five models (AdaBoostM1, BN, KNN, LR, SVM) across eight key evaluation metrics. The color gradient (from blue to red) represents the magnitude of the metric values, with the color bar on the right denoting the scale. AdaBoostM1, adaptive boosting algorithm M1; BN, Bayesian network; KNN, k-nearest neighbors; LR, logistic regression; SVM, support vector machine. [file Image_2.jpg]

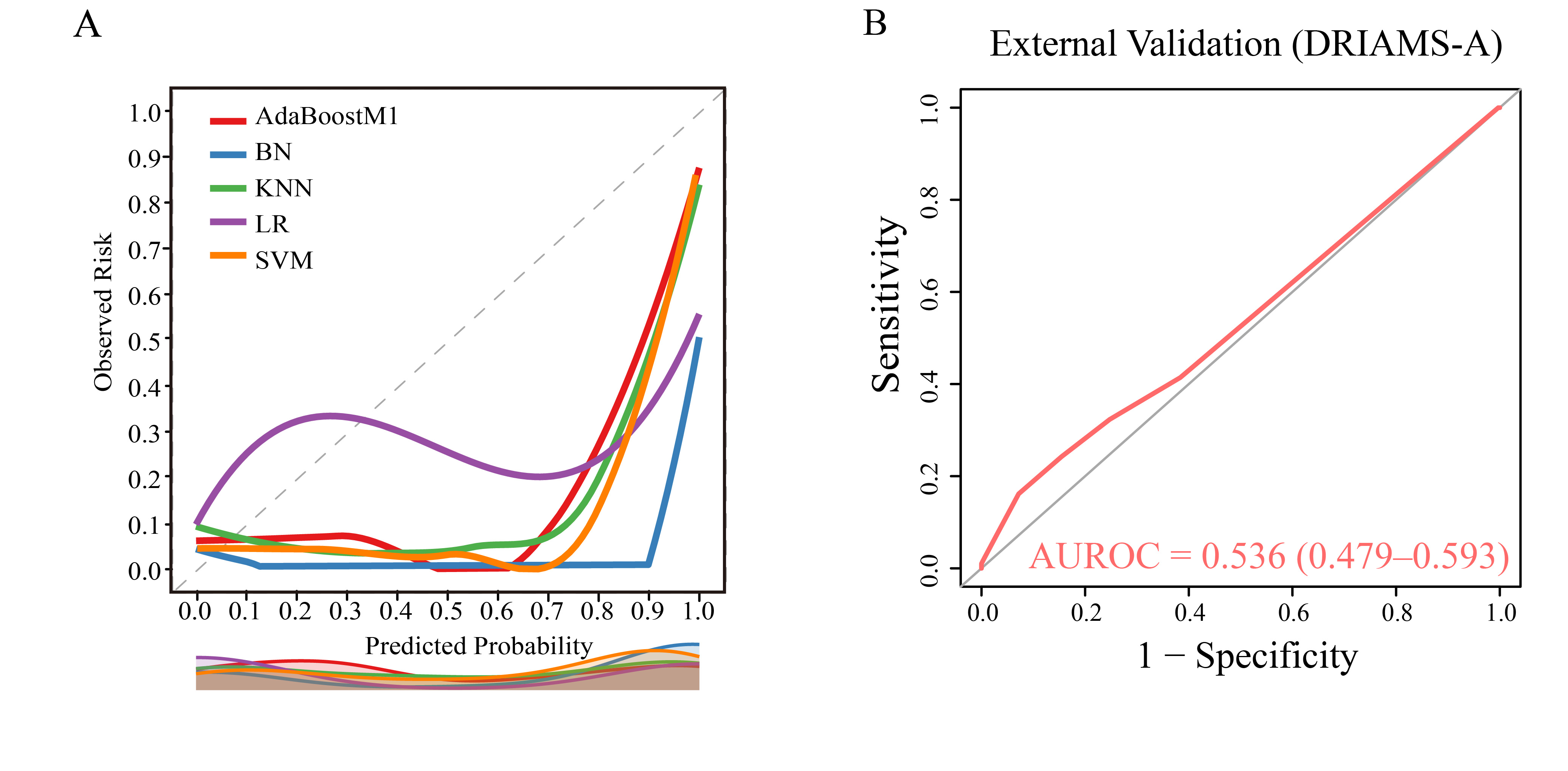

Supplement: Supplementary Figure 3 — Model calibration and spatial external validation. (A) Calibration curves for five models (AdaBoostM1, BN, KNN, LR, SVM) on the temporal external validation set. The black dashed line represents ideal calibration; shaded areas denote 95% CI. (B) ROC curve of the KNN classifier on the DRIAMS-A spatial external validation cohort. AdaBoostM1, adaptive boosting algorithm M1; BN, Bayesian network; KNN, k-nearest neighbors; LR, logistic regression; SVM, support vector machine; AUROC, area under the receiver operating characteristic curve; CI, confidence interval. [file Image_3.jpg]
