# Supplementary material for: Rapid prediction of vancomycin-resistant Enterococcus faecium using MALDI-TOF mass spectrometry and machine learning
Source: Front Microbiol. 2026 Jun 16;17:1789841. doi: 10.3389/fmicb.2026.1789841 (PMC13314634; doi:10.3389/fmicb.2026.1789841)
Supplement: Supplementary file 4 [file Table_1.docx]

**Supplementary Table 1.** Optimal hyperparameters for the KNN model.

| **Hyperparameter** | **Symbol** | **Optimal Value** | **Description** |
| --- | --- | --- | --- |
| Number of neighbors | *k* | 10 | *k* for k-NN classifier |
| Distance | *d* | 2.0 | Euclidean distance |
| Kernel | — | rectangular | Neighbor weighting kernel |
| Scale | — | TRUE | Feature scaling enabled |
| Store model | — | FALSE | Do not retain model |

**Supplementary Table 2.** Top influential feature bins.

| **Number** | **Feature bin** | **Number** | **Feature bin** | **Number** | **Feature bin** | **Number** | **Feature bin** |
| --- | --- | --- | --- | --- | --- | --- | --- |
| **1** | 10080-10085 | **11** | 3460-3465 | **21** | 4410-4415 | **31** | 6605-6610 |
| **2** | 2670-2675 | **12** | 3495-3500 | **22** | 5040-5045 | **32** | 6630-6635 |
| **3** | 2980-2985 | **13** | 3620-3625 | **23** | 5095-5100 | **33** | 6635-6640 |
| **4** | 3020-3025 | **14** | 3640-3645 | **24** | 5125-5130 | **34** | 6700-6705 |
| **5** | 3165-3170 | **15** | 3645-3650 | **25** | 5245-5250 | **35** | 6705-6710 |
| **6** | 3170-3175 | **16** | 3650-3655 | **26** | 5250-5255 | **36** | 6910-6915 |
| **7** | 3300-3305 | **17** | 3655-3660 | **27** | 5935-5940 | **37** | 6920-6925 |
| **8** | 3310-3315 | **18** | 3665-3670 | **28** | 6535-6540 | **38** | 7305-7310 |
| **9** | 3440-3445 | **19** | 3670-3675 | **29** | 6540-6545 | **39** | 7550-7555 |
| **10** | 3445-3450 | **20** | 3685-3690 | **30** | 6565-6570 | **40** | 8330-8335 |

**Supplementary Table 3.** Performance of machine learning models on the training set. Abbreviations: LR, logistic regression; SVM, support vector machine; BN, Bayesian network; AdaBoostM1, Adaptive Boosting Algorithm M1; KNN, K-nearest neighbors.

| **Model** | ***AB*_score_** | **AUC** | **Brier score** |
| --- | --- | --- | --- |
| AdaBoostM1 | 0.96 | 0.97 | 0.06 |
| BN | 0.96 | 0.98 | 0.06 |
| KNN | 0.95 | 0.96 | 0.05 |
| LR | 0.95 | 0.97 | 0.06 |
| SVM | 0.97 | 0.98 | 0.04 |

**Supplementary Table 4.** Performance of machine learning models on the temporal external validation set. Abbreviations: LR, logistic regression; SVM, support vector machine; BN, Bayesian network; AdaBoostM1, Adaptive Boosting Algorithm M1; KNN, K-nearest neighbors.

| **Model** | ***AB*_score_** | **AUC** | **Brier score** |
| --- | --- | --- | --- |
| AdaBoostM1 | 0.86 | 0.90 | 0.17 |
| BN | 0.78 | 0.95 | 0.39 |
| KNN | 0.84 | 0.90 | 0.21 |
| LR | 0.79 | 0.84 | 0.26 |
| SVM | 0.81 | 0.89 | 0.27 |

**Supplementary Table 5.** Comparison between Sun 2026 (this work) and Weis 2022. Abbreviations: *E. faecium*, *Enterococcus faecium*; LR, Logistic Regression; SVM, Support Vector Machine; BN, Bayesian Network; AdaBoostM1, adaptive boosting algorithm M1; KNN, K-Nearest Neighbors; LightGBM, Light Gradient Boosting Machine; MLP, multilayer perceptron; XGBoost, eXtreme Gradient Boosting; AUROC, area under the receiver operating characteristic curve; AUPRC, area under the precision-recall curve; DCA, decision curve analysis; SHAP, SHapley Additive exPlanations.

|  | **Sun *et al*. 2026** | **Weis *et al*. 2022** |
| --- | --- | --- |
| **Research subject** | Vancomycin-resistant *E. faecium* | Resistance of *Escherichia coli* and *Klebsiella pneumoniae* to ceftriaxone; and resistance of *Staphylococcus aureus* to oxacillin |
| **Machine learning algorithm** | LR, SVM, BN, AdaBoostM1, and KNN | LR, LightGBM and MLP |
| **Key performance**  **metrics** | AUROC, AUPRC, DCA, calibration curves, accuracy, precision, recall and F1-score | AUROC and AUPRC |
| **Validation** | Internal validation and temporal validation | Cross-site validation and site-specific validation |
| **Feature selection algorithm** | XGBoost-RFE | Full preprocessed spectrum |
| **Feature**  **Interpretation** | SHAP value | SHAP value |

1. **Research subject:**
   1. **Pathogen and antibiotic specificity:**

This study investigates the application of machine learning integrated with mass spectrometry to predict the resistance of *E. faecium* to vancomycin. In contrast, the study by Weis *et al.* examines the resistance of *Escherichia coli* and *Klebsiella pneumoniae* to ceftriaxone, and the resistance of *Staphylococcus aureus* to oxacillin.

- 1. **Geographic and Epidemiological Correlation:**

Our clinical isolates (n=268) from Chinese hospitals and the concurrent inclusion of the European DRIAMS-A dataset provided a unique framework for assessing geographic generalizability. Consistent with Weis et al., who demonstrated that cross-site validation generally underperforms relative to within-site validation across European hospitals, we observed that external-only models failed to exceed chance-level discrimination (AUROC ≈ 0.50). Conversely, hybrid local-external strategies achieved clinically acceptable performance (AUROC > 0.80), indicating that strategic integration of large external datasets can partially mitigate domain shifts for sites with limited local data, albeit with variable effectiveness across geographic contexts. These findings suggest that geographic variation in bacterial epidemiology and antimicrobial resistance mechanisms produces distinct mass spectrometry profiles, underscoring the necessity for region-specific model development while validating pooled training as a pragmatic alternative.

- 1. **Spectral Stability Across Variable Incubation Durations**

Unlike Weis et al., who analyzed spectra from 24-hour cultures exclusively, our study assessed the temporal robustness of 24-hour-derived models by validating them against 48-hour incubation spectra. This extension addresses whether spectral signatures remain stable across prolonged culture durations, offering broader operational flexibility for clinical laboratories with variable incubation schedules.

1. **Analytical Methods**
   1. **Model Development**

In terms of feature selection, our study leveraged the XGBoost algorithm to identify 40 key feature peaks, which delivered optimal accuracy. This focused approach contrasts with Weis *et al*.'s methodology, which utilized preprocessed full-spectrum data encompassing approximately 6000 features. Our research developed and evaluated five machine learning models: LR, SVM, BN, AdaBoostM1, and KNN. Notably, the KNN model exhibited superior and robust performance, achieving AUROC values ranging from 0.82 to 0.90 and AUPRC values ranging from 0.64 to 0.82 on the temporal validation set. In comparison, Weis *et al*. employed algorithms including LR, LightGBM, and MLP, with LightGBM and MLP demonstrating the highest overall performance.

- 1. **Validation**

To ensure model reliability, we implemented rigorous internal validation through 5-fold cross-validation and further assessed generalizability via temporal external validation (2025 holdout set) and spatial external validation (DRIAMS-A dataset). Unlike Weis et al., whose within-site and cross-site validation was confined to multiple European hospitals, our design explicitly tested model performance on an independent, geographically distinct external cohort, thereby providing a more stringent evaluation of geographic transportability.

- 1. **Performance Evaluation**

In addition to AUROC and AUPRC reported in prior work, we calculated confusion matrix-derived metrics (accuracy, precision, recall, and F1 score) to capture threshold-specific performance. Moving beyond discriminative ability, we assessed model calibration using calibration curves and evaluated clinical utility via decision curve analysis (DCA), thereby providing a comprehensive appraisal of both statistical reliability and practical clinical applicability.

- 1. **Feature selection algorithm**

The original spectra were discretised into 5-Da intervals (2,000-20,000 Da) with subsequent selection of the 40 most predictive features for modelling. SHAP (SHapley Additive exPlanations) values were leveraged to enable dual-level interpretation through global and local analytical frameworks. Conversely, Weis *et al*. implemented 3-Da spectral binning (2,000-20,000 Da) while similarly applying SHAP for feature importance assessment. Their approach, however, focused predominantly on aggregate feature prioritisation, omitting comparably detailed visual elucidation at the individual sample level.

1. **Key Innovations Summarised**

The study by Weis *et al*. has been validated in clinical settings through the integration of detailed clinical case data and has demonstrated potential for incorporation into decision-making processes; however, this study currently focuses primarily on the technical development, evaluation, and interpretation of the prediction model; yet, it has not yet been integrated with clinical decision-making systems. Future work will build upon this high-performance model to facilitate its integration and application within clinical workflows.
